# Host species shape the community structure of culturable endophytes in fruits of wild berry species (*Vaccinium myrtillus* L., *Empetrum nigrum* L. and *Vaccinium vitis-idaea* L.)

**DOI:** 10.1093/femsec/fiab097

**Published:** 2021-07-12

**Authors:** Minh-Phuong Nguyen, Kaisa Lehosmaa, Françoise Martz, Janne J Koskimäki, Anna Maria Pirttilä, Hely Häggman

**Affiliations:** Ecology and Genetics Research Unit, University of Oulu, FI-90014, P.O. Box 3000, Oulu, Finland; Ecology and Genetics Research Unit, University of Oulu, FI-90014, P.O. Box 3000, Oulu, Finland; Natural Resources Institute Finland, Production Systems, Ounasjoentie 6, FI-96200, Rovaniemi, Finland; Ecology and Genetics Research Unit, University of Oulu, FI-90014, P.O. Box 3000, Oulu, Finland; Ecology and Genetics Research Unit, University of Oulu, FI-90014, P.O. Box 3000, Oulu, Finland; Ecology and Genetics Research Unit, University of Oulu, FI-90014, P.O. Box 3000, Oulu, Finland

**Keywords:** food microbiology, endophytic communities, microbial ecology, host-specific, metabolic crosstalk, soluble phenolics

## Abstract

Wild berries are interesting research subjects due to their rich sources of health-beneficial phenolic compounds. However, the internal microbial communities, endophytes, associated with the wild berry fruits are currently unknown. Endophytes are bacteria or fungi inhabiting inside plant tissues, and their functions vary depending on the host species and environmental parameters. The present study aimed to examine community composition of fungal and bacterial endophytes in fruits of three wild berry species (bilberry *Vaccinium myrtillus* L., lingonberry *Vaccinium vitis-idaea* L. and crowberry *Empetrum nigrum* L.) and the effects of host plant species and their growth sites on shaping the endophytic communities. We found that the endophytic community structures differed between the berry species, and fungi were predominant over bacteria in the total endophytic taxa. We identified previously unknown endophytic fungal taxa including *Angustimassarina*, *Dothidea*, *Fellozyma*, *Pseudohyphozyma*, *Hannaella coprosmae* and *Oberwinklerozyma straminea*. A role of soluble phenolic compounds, the intracellular components in wild berry fruits, in shaping the endophytic communities is proposed. Overall, our study demonstrates that each berry species harbors a unique endophytic community of microbes.

## Teaser

Fruits of wild berry species bilberry, crowberry and lingonberry in Northern Europe harbor host-specific endophytic communities.

## INTRODUCTION

Endophytes are mainly bacteria and fungi that inhabit inside plant tissues for all or a part of their lifetime without creating symptoms of disease (Hardoim *et al*. 2015). Various plant parts are colonized by different endophytic communities within a species or plant individual (Bodenhausen, Horton and Bergelson 2013; Ottesen *et al*. 2013) and these communities can originate from diverse sources. Root endophytes are likely to colonize the plant from the soil environment, while endophytes of the above-ground plant tissues can originate from the rhizosphere, phyllosphere or from the seeds (Hardoim *et al*. 2015). This indicates that host genotype and environmental origin have prominent roles in shaping the endophytic communities of specific plant tissues.

Both vertical (via seeds and pollen) and horizontal (via soil, air, water and insects) transmission have been reported for endophytes (Compant *et al*. 2011; Hardoim *et al*. 2012; Hodgson *et al*. 2014; Lòpez-Fernàndez *et al*. 2017). Seed-borne endophytes passing to the next generation play important roles in host growth and defence and provide offspring with valuable symbionts (Shahzad *et al*. 2018). Horizontally transmitted endophytes are reported as host genotype-specific (Rajala *et al*. 2013; Unterseher *et al*. 2013). Among the two transmission modes, the predominant one depends on the identity of endophytes (Rodriguez *et al*. 2009).

The metabolic crosstalk between endophytic microbes and their host has recently been discussed (Lòpez-Fernàndez *et al*. 2016). There is evidence that these microbes can influence their host by releasing bioactive compounds or by modifying nutrient balance and plant fitness to increase tolerance to abiotic and biotic stresses (Pacifico *et al*. 2019). They can also modify the secondary metabolites of inoculated plants (Lòpez-Fernàndez *et al*. 2016; Yang *et al*. 2016; Pan *et al*. 2020). On the other hand, the host plant can shift the concentration of specific metabolites to favor colonization of specific endophytes (Lòpez-Fernàndez *et al*. 2016).

Studies on endophytes can open possibilities to harness beneficial endophytes for various biotechnological applications. The most important application field of endophytes is in pharmaceuticals, where endophytic compounds possessing bioactive characteristics, such as anticancer, antioxidant, antifungal and antibacterial ones are used as potential drug sources. For example, the endophyte *Trametes hirsuta* from *Podophyllum hexandrum* can produce podophyllotoxin, an antioxidant and anticancer compound (Puri *et al*. 2006). In agriculture, plant growth-promoting endophytes, or their compounds, reduce inputs of pesticides and fertilizers on fields, contributing to eco-friendly crop production (Rai *et al*. 2014). Moreover, enzymes such as cellulases, pectinases, proteases and xylanases produced by endophytic microbes have potential for various industrial, agricultural and medicinal applications (Alvin *et al*. 2014).

Wild plant species are considered interesting subjects for endophyte studies because of their high genetic variability and often harsh growth conditions. Plants living in the wild are expected to harbor a wider range of microbial taxa than their related commercial species, including the taxa that are beneficial for their survival in demanding habitats (Ofek-Lalzar *et al*. 2016; Llorens *et al*. 2019). For example, endophytes are shown to enable the host plant to live under extreme temperatures (Marquez *et al*. 2007; Subramanian *et al*. 2015). Although wild plants are potent reservoirs of new beneficial endophytes, most analyses on endophytic communities have been performed on agricultural crops or model plant species (Lugtenberg, Caradus and Johnson 2016; Gdanetz and Trail 2017).

Similarly, endophytic communities in plant reproductive organs are less studied compared to other plant parts. Examples include grape berries (Dugan, Lupien and Grove 2002), cranberry ovary (Tadych *et al*. 2012), coffee berries (Vega *et al*. 2008), cucurbits fruits (Glassner *et al*. 2015), strawberry seeds (Kukkurainen *et al*. 2005), apple, pear (Glushakova and Kachalkin 2017) and papaya fruits (Krishnan *et al*. 2012). The reproductive parts of these plants were likely all studied due to their importance for food industry, while those of wild species are barely touched. However, studying these organs is also essential due to their role in genetic transmission to offspring.

In the present study, we focused on fruits of wild bilberry, crowberry and lingonberry, all well-known natural resources in Northern Europe (Heinonen 2007; Miina, Hotanen and Salo 2009). Research on endophyte communities of northern wild berry fruits is urgently needed due to their importance as functional and therapeutic foods (Zafra-Stone *et al*. 2007; Manganaris *et al*. 2014). These berry species grow as mixed populations in the same habitats across forests in Northern Europe, which provides an excellent scenery to study the influence of host species and environment on endophytic communities. Moreover, these wild berries survive in cold climate conditions, where some endophytic species could support host survival in the harsh conditions.

Although the phenolic compounds of wild berries have been largely studied, research on their microbiomes is lacking. To the best of our knowledge, the present study is the first one focusing on endophytes in lingonberry (*Vaccinium vitis-idaea* L.). In a previous study on black crowberry leaves (*Empetrum nigrum* L.), mainly unknown endophytic taxa were found (Tejesvi *et al*. 2016). In the case of bilberry, only two reports focusing on specific endophytes isolated from twigs and leaves exist (Fisher, Anson and Petrini 1984; Koskimäki *et al*. 2009). To our knowledge, no comprehensive analysis of endophytic communities in the fruits of the three wild berry species is available. Thus, the aim of the present study was to examine the community composition of culturable fungal and bacterial endophytes in fruits of bilberry, lingonberry and crowberry, and investigate the effects of host plant species and their growth sites on shaping the communities. Moreover, we were interested whether the host phenolic compounds could explain the endophytic composition.

Based on the existing literature, we hypothesized that both the berry species and the environment are able to shape the fruit endophytic community (Hardoim *et al*. 2015; Pacifico *et al*. 2019). Moreover, we assumed that the composition of berry fruit phenolics describe endophytic community structure, as a strong metabolic cross-talk exists between host and endophytes (Lòpez-Fernàndez *et al*. 2016; Yang *et al*. 2016; Pan *et al*. 2020).

## MATERIALS AND METHODS

### Sample collection

Bilberry, crowberry and lingonberry fruits used in the study were collected from three sites in the subarctic Oulu region, Northern Finland (Appendix S2: Table S1, Supporting Information and Appendix S1: Figure S1, Supporting Information). The minimum distance between sites was 400 m and the maximum one was 950 m. The sites were selected since they contained mixed populations of the three berry species, which ensured the comparability between the isolated endophyte communities. The growth site O2 was the most undisturbed forest among the three selected sites; it had high density and diverse plant species. The sites O1 and O3 were similar in the environmental conditions; they had a lower vegetation density and diversity compared to the site O2, and they were more affected by urban activities.

We used forceps to collect the berry fruits at the stage of full maturity. The fruits were preserved in 50 mL falcon tubes and were kept on ice (+4°C) for transportation and further processing. A total of 30–50 fruits (3–10 g depending on the fruit sizes) from the collected samples were used immediately for endophyte isolation. The remaining samples were stored at −80°C and used for phenolic compounds analysis by LC/MS.

### Isolation of the endophytes from the wild berry fruits

The fruits were pooled and surface sterilized to remove epiphytes and contaminants from the skin of the berries. The samples were washed three times with deionized water to remove dust and debris, surface-sterilized with 70% ethanol for 30 s and then with 3% (w/v) calcium hypochlorite containing 0.125% (w/v) Tween-80 for 5 min. Finally, the samples were rinsed five times in sterile milli-Q water and dried on sterilized filter papers to absorb excess water. Gentle shaking was applied to all steps of the sterilization procedure.

Sterilized berries were crushed in sterile mortars with pestles and mixed with 1 mL of sterile Phosphate Buffered Saline (PBS, pH 7.4). All extracts were spread on four different culture media: potato dextrose agar (PDA) containing 100 mg L^-1^streptomycin medium, 2% water agar, M9 and 1/10 diluted 869 media. PDA and 1/10 869 media were selected, because these rich media support maximal endophyte recovery in both quantity and type (Eevers *et al*. 2015; Singh *et al*. 2017). Water agar and M9 media were selected to support the growth of slow-growing endophytes. PDA and water agar were used to grow fungi while 1/10 diluted 869 and M9 were for bacteria. Plates were dried for 15 min aseptically and sealed by parafilm. The plates were divided in two identical sets; one set was incubated at +21°C while another was kept at +12°C. The plates were checked every day and each endophyte was immediately transferred to a fresh plate to obtain a pure culture. Stock cultures were made in 30% glycerol for fungi, in 50% glycerol for endophytic bacteria and stored at −80°C.

To test the efficacy of the method, three sterilized berries for each sample were imprinted for 30 min on the 869 rich medium plates (Eevers *et al*. 2015), and the fruits were removed from the plates. Also, 250 µL of the last rinsing water was spread on the 869 plates. The control plates were incubated in the same conditions as the isolated plates to confirm the absence of any microbial growth after sterilization.

### Morphological and molecular identification of the endophytes

For each berry sample, we grouped their endophytes into morphotypes based on the morphological details. Specifically, endophytic fungi were clustered by the shape, size, color, texture of the colony in both front and back sides. Bacterial endophytes were grouped based on the colony color, form, elevation, margin, size, surface, opacity and texture. Then for each morphotype of each berry sample, we selected one representative to sequence its marker gene.

We applied the heat lysis method (Ganguly *et al*. 2005) with modification to sequence our endophytic samples. The endophytic samples were collected and mixed with 200 µL of lysis buffer (10 mM Tris-HCl, 0.1 mM EDTA, 0.1% Triton X-100, pH 8) and stored at −20°C for amplification of the marker genes and sequencing. Upon amplification, 20 µL of lysis mixture of each sample was heated at +98°C for 10 min by the Master Gradient 89157 PCR machine. Then the mixture was centrifuged at 2000 x *g* for 5 min to separate the supernatant and debris. A total of 2 µL of the supernatant (5-20 ng DNA) was used as the template in 20 µL of PCR reaction containing 1X Phusion HF buffer, 1.5 mM MgCl_2_, 200 µM dNTP, 0.25 µM of each primer and 0.025 U of Phusion polymerase. Reaction conditions consisted of an initial heating at +98°C for 1 min, followed by 30 cycles of +98°C for 10 s, +55°C for 20 s, +72°C for 20 s and a final extension at +72°C for 7 min. The forward primer ITS7bF (5’-GTGARTCATCGAATCTTTG-3’) and the reverse primer ITS4R (5’-TCCTCCGCTTATTGATATGC-3’) were used to amplify ITS2 region of fungi (Ihrmark *et al*. 2012). The forward primer 515F (5’-GTGYCAGCMGCCGCGGTAA-3’) and the reverse primer 926R (5’- CCGYCAATTYMTTTRAGTTT-3’) were used to amplify V4 and V5 region of the 16S ribosomal rRNA gene of bacteria (Walters *et al*. 2016).

For the samples that could not be amplified successfully with the heating method mentioned above, the genomic DNA was isolated with E.Z.N.A. ® SP Plant DNA Kit. Specifically, 40–90 mg of fresh culture of each sample was collected into a 2 mL sterilized tube. Collection of samples was done carefully to pick as much microbial culture as possible without picking agar. Then the tubes were frozen in liquid nitrogen, and the samples were homogenized at 25 Hz for 90 s with sterilized 5 mm stainless steel beads by the Tissue Lyzer. Homogenized samples were kept on ice and immediately extracted following the protocol of the E.Z.N.A. ® SP Plant DNA Kit. For yeast- and bacteria-type endophytes, the cell culture was washed with 1 mL sterile water and then centrifuged to remove extracellular polysaccharides before their genomic DNA were extracted by the kit. Concentrations of the extracted DNA ranged from 3 to 100 ng/µL depending on the samples being either fungi or bacteria. For the marker gene amplification, 6–100 ng of the DNA was used as the template.

PCR products (20–50 ng) were cleaned by ExoI-FastAP kit (Thermo scientific, Vilnius, Lithuania) according to manufacturer's instructions. For those PCR products that had multiple bands or primer dimers visualized on agarose gel, DNA bands of interest were extracted by the Montage DNA Gel Extraction Kit following the protocol of (Jaakola *et al*. 2004) with modifications. Specifically, 15 µL of each PCR product was mixed with 1.8 µL of 6X Orange Loading Dye (Thermo Fisher Scientific, Vilnius, Lithuania) and loaded into a 1%-ethidium bromide agarose gel in 1X Tris Acetate-EDTA buffer (included in the Montage DNA Gel Extraction Kit). Gel electrophoresis conditions were 120 V for 50 min. Each DNA band of interest was sliced with a new surgical blade-11 (Mediq, Osaka, Japan) and purified according to manufacturer's instructions. The collected DNA was precipitated with 1/10 V of 3 M sodium acetate and 2.5 V of absolute ethanol for at least 1 h at −80°C,  followed by two washing steps with 1 mL 70% ethanol.

Either 2 µL of ExoI-FastAP purified PCR product or 3–10 ng of gel-purified PCR product was used as the template in a 10 µL sequencing reaction and sequenced according to manufacturers’ instructions (BigDye Terminator v3.1 Cycle Sequencing Kit, Thermo scientific).

Sequencing products were cleaned by gel filtration method. Specifically, dry Sephadex® G-50 (Sigma, Saint Louis, USA) was loaded onto 96-well MultiScreen HV plate using the 45 µL Column Loader. The gel was allowed to imbibe 300 µL of sterile milli-Q water overnight at +4°C,  after which it was packed by centrifugation at 910 x *g* for 5 min. Then, 20 µL of sterile water was added to each sequencing product to reach a total volume of 30 µL. The sequencing products were carefully loaded into the center of each well and collected into a barcoded 96-well plate by centrifugation at 910 x *g* for 5 min and analysed by the ABI 3730 DNA Sequencing System.

### Taxonomic assignment

#### Quality trimming of the sequencing data and generating of the contigs

*Tracetuner* 3.0.6beta (Denisov, Arehart and Curtin 2004) was used to call the bases and assign the corresponding quality value from the chromatogram files generated by the ABI 3730 sequencer. It resulted in a FASTA and a QUAL file for each chromatogram file, which were used as the inputs for the next quality trimming step. To trim low quality bases from the sequences, *LUCY* 1.20 (Chou and Holmes 2001) and custom python scripts were used. Since *LUCY* 1.20 tagged the good quality ranges of the sequences instead of clipping the sequences, the custom python scripts were used to trim input DNA sequences based on the output information of *LUCY*. First, a FASTQ file was generated from the FASTA and QUAL files for each sequence. Then, the generated FASTQ files were trimmed based on the tagging information of *LUCY*. *Biopython* 1.75 was the primary Python package used in our custom scripts, and all the scripts were run in the *python* 3.7.3 environment.

All trimmed forward and reverse FASTQ files were concatenated into two single files corresponding to forward and reverse sequences. The contigs for each sequence were generated from both files by the command line *make.contigs* in the *mothur* environment v.1.30.0. Then the contigs were analyzed using BLAST reference databases. The sequence data have been submitted to the GenBank databases under accession number MW433932–MW433974 (for bacteria) and MW448745–MW449082 (for fungi).

#### Aligning the contigs against bacterial and fungal databases

For bacterial sequences, the SILVA Release 138 SSU Ref NR 99 (truncated) was used as the reference (Quast *et al*. 2013). For fungal sequences, Full UNITE+INSD dataset for Fungi (created on 04.02.2020) was used as the reference (Nilsson *et al*. 2019). BLAST databases were built from these references by the command line version of *BLAST* (called *BLAST*+ v.2.10.0). The endophytic contigs were aligned with the BLAST databases, and the best hit for each contig was selected based on the parameters of the alignment (score, e-value, coverage, mismatch and gap). Sequences with the similarity higher than 97%, matched with the same best hit, and had the same colony morphology were considered the same species.

### Phenolic compound analysis

For soluble phenolic extraction, about 100 berries/sample were pooled, freeze dried (Labconco, FreeZone plus 4,5l Cascade) and ground up to fine powder using a bead homogenizer (Precellys 24 dual, Bertin). Soluble phenolics were extracted in [methanol: H_2_O (1:1), 0.1% HCl] as described previously (Häkkinen *et al*. 1999). Test extractions with the antioxidant butylated hydroxytoluene BHT (1 mg mL^-1^) and 0.1% HCl showed identical patterns of soluble phenolic compounds, therefore, instead of BHT, 0.1% HCl was added to improve anthocyanin (ACN) stability. A total of 20 mg of dry powder was extracted with 0.6 mL [methanol: H_2_O (1:1), 0.1% HCl] by 5 min of sonication, followed by shaking for 10 min in the dark. Samples from each berry species and location were extracted three times (three technical replicates). Samples were analyzed immediately after extraction by UPLC-DAD-ESI-MS/MS (Nexera2, LCMS-8040, Shimadzu, Kyoto, Japan) using a Luna 5 µm C18(2) 100 Å, 250×3 mm column (Phenomenex, Torrance, USA) and a C18 guard column with solvent A (10% methanol and 0.1% formic acid) and solvent B (98% methanol, and 0.1% formic acid) and the following gradient: 0–2 min of 5% B, 15 min of 13% B, 30 min of 40% B, 40–50 min of 100% B (flow 0.3 mL min^-1^, column oven 40°C). The MS conditions were as follows: nebulizing gas (N_2_) 3 L min^-1^, drying gas (N_2_) 15 L min^-1^, desolvation line 250°C, heat block temperature 400°C, interface voltage 4.5 kV.

Quantification was done using MS detection with single ion monitoring and multiple reaction monitoring (MRM) (phenolic acids, flavonoids and proanthocyanidins i.e. PAs and anthocyanins i.e. ACNs) in negative or positive modes (Appendix S2: Table S2, Supporting Information; Määttä-Riihinen *et al*. 2005; Tian *et al*. 2005; Ek *et al*. 2006). Several authentic standards were used for quantification depending on the compound. Calibration curves for ACNs and PAs were calculated using spiked samples. Standards were purchased from Extrasynthese (cyanidin 3-O- glucoside, procyanidins A2, procyanidins B2, hyperoside, kaempferol 3-O- glucoside, gallic acid) and Sigma-Adrich (catechin, 3-O-chlorogenic acid (CGA), protocatechuic acid (PCA)). Unknown compounds were quantified using catechin. The precision of the concentrations, calculated from the relative standard deviation of the three technical replicated extractions, was <8.7% for compounds more abundant than 0.01 mg g^-1^ DW (10.0% for compounds more abundant than 0.005 mg g^-1^ DW).

### Statistical analyses

Endophytic data was in the presence-absence (1/0) format, and all statistical analyses were selected regarding this format. The analyses and visualization were carried out in R version 4.0.3 (R Core Development Team 2019) using packages *vegan* v.2.5–6, *stats* v.3.6.0*, car* v.3.0–8, *betapart* v.1.5.1 and *indicspecies* v.1.7.9 (Baselga and Orme 2012; De Cáceres and Legendre^2009^; Fox and Weisberg 2019; Oksanen *et al*. 2019).

Differences in numbers of observed species (taxonomic richness) among the berry species and growth sites were tested with analysis of variance (ANOVA) using *aov* function of *stats* package. Prior to ANOVA analysis normal distribution was tested with *Levene´s Test* of *car* package.

In order to explore the endophytic community structure (β diversity), we computed a pairwise Raup–Crick dissimilarity matrix (βrc) using the *raup*–*crick* function provided by Chase *et al*. (2011). This function measures β diversity while controlling effects of differences in α diversity (richness) on β diversity since it uses a null-modelling approach. We also tested another classic measure of β diversity for presence/absence data, Sørensen (Anderson *et al*. 2011). The pairwise Sørensen dissimilarity matrix was computed by the *beta.pair* function of *betapart* package (Baselga *et al*. 2018). The structure of the endophytic community was visualized with non-metric multi-dimensional scaling (NMDS) using *metaMDS* function of *vegan* package.

Statistical differences in endophytic community composition among berry species and growth sites were examined using one-way Permutational Multivariate Analysis of Variance (PERMANOVA with 9999 permutations) using *adonis* function. The multivariate homogeneity of group dispersions was tested using *betadisper* function to examine whether the dispersion of any group was significantly different from the others. The *P*-value (> 0.05) from the test indicates the balance of group dispersion (Table 1). Hierarchical cluster analysis (HCA) was carried out using the UPGMA agglomerative clustering method in *hclust* function of *stats* package to reveal the structure residing in a dataset (Ramette 2007). The results with Sørensen index were similar to those of Raup–Crick's, and in the main text we only report results of Raup–Crick's index. The results with Sørensen index are shown in the supplement.

**Table 1.** Multivariate dispersion analysis and PERMANOVA of category effects on microbial diversity pattern based on Raup–Crick and Sørensen dissimilarity matrixes. (*) and (**) indicate *P* < 0.05 and *P* < 0.01, respectively.

			Fungi + Bacteria			Fungi		
		Factor	F	*P*		F	*P*	
Multivariate dispersion	Raup–Crick	Berry	2.508	0.162		3.278	0.109	
		Location	0.464	0.649		0.436	0.666	
	Sørensen	Berry	0.676	0.543		0.445	0.661	
		Location	0.396	0.689		0.385	0.696	
PERMANOVA	Raup–Crick	Berry	2.562	0.010	*	2.272	0.032	*
		Location	0.464	0.912		0.532	0.852	
	Sørensen	Berry	1.408	0.003	**	1.422	0.006	**
		Location	0.930	0.695		0.956	0.582	

To further evaluate changes in β diversity, we assessed the relative contribution of the components of beta diversity (i.e. turnover and nestedness) for all three hosts with *beta.multi* function of *betapart* package using the Sørensen dissimilarity index (Baselga *et al*. 2018). This function partitions the changes in beta diversity into the nestedness and turnover components. Turnover component means species replacement among the samples while nestedness means that some species are lost (i.e. low diversity samples are nested subsets of high-diversity samples). We then obtained the proportion of turnover component to overall Sørensen dissimilarity to represent the relative contribution of overall β diversity: β_ratio_ = β_SIM_ ÷ β_SOR_. β_ratio_ < 0.5 indicates that β diversity is driven primarily by nestedness while β_ratio_ > 0.5 indicates the predominant role of turnover.

To identify indicator endophytic species for each group (berry or growth site), we calculated the Indicator Value (IndVal) index to measure the association between a species and a group with *multipatt* function of the *indicspecies* package.

We standardized and computed the Euclidean dissimilarity matrix for the phenolic compound data using *vegdist* function. Variation of phenolic compounds were presented with principal component analysis (PCA) with *rda* function. The most important principal components (PCs) were determined by the broken-stick model in which the eigenvalues of PCs larger than the values given by the broken stick model were selected (Jackson 1993). Multivariate homogeneity of group dispersions and statistical differences among the berry species and growth sites of the phenolic compositions were assessed as described above.

Quantifications of collinearity of phenolic variables were done using pairwise correlation coefficient (r) with *cor* function of *stats* package. It was essential to detect variables collinear and deal with it because collinearity can lead to the wrong identification of relevant predictors in statistical models (Dormann *et al*. 2013). After detecting collinearity, we used PCA for clustering variables to remove correlations in a phenolic set and to reduce collinearity (Dormann *et al*. 2013). For each PC axis, variables with absolute loadings larger than 0.43 formed one cluster, the threshold was chosen to ensure that each cluster contained unique variables. Once the cluster was identified, we performed PCA based on the variables in the cluster and used the resulted PC1 as the ‘cluster scores’. A new matrix containing all the ‘cluster scores’ and variables that did not fall into any clusters were standardized and used in further analysis.

We applied dbRDA to examine the relative contributions of each of the collinearity-removal phenolic variables on the endophytic community assemblages based on βrc dissimilarity by using *capscale* function of *vegan* package. To select the most influential phenolic variables we used *ordistep* function to exclude variables with no significant contribution (*P* > 0.05). The significance of the dbRDA model and each predictor was assessed using permutation tests with *anova* function in *vegan*.

## RESULTS

### Taxonomic composition

Fungi were isolated from all the samples. Bacteria were cultivated from most of the samples except lingonberry at site O3. Altogether, 338 fungi and 43 bacteria were selected based on the morphology screening, and they were sequenced with marker-gene primers. After aligning forward and reverse sequences to construct each contig, the average length of the amplified bacterial 16S rRNA fragment was 394 bp (ranging from 328 to 414 bp), and that of fungal ITS rRNA fragment was 271 bp (ranging from 187 to 371 bp). The endophytes were clustered into 172 fungal (Appendix S1: Figures S2–S4, Supporting Information) and 18 bacterial taxa based on alignment with reference databases and colony morphology.

**Figure 1.**
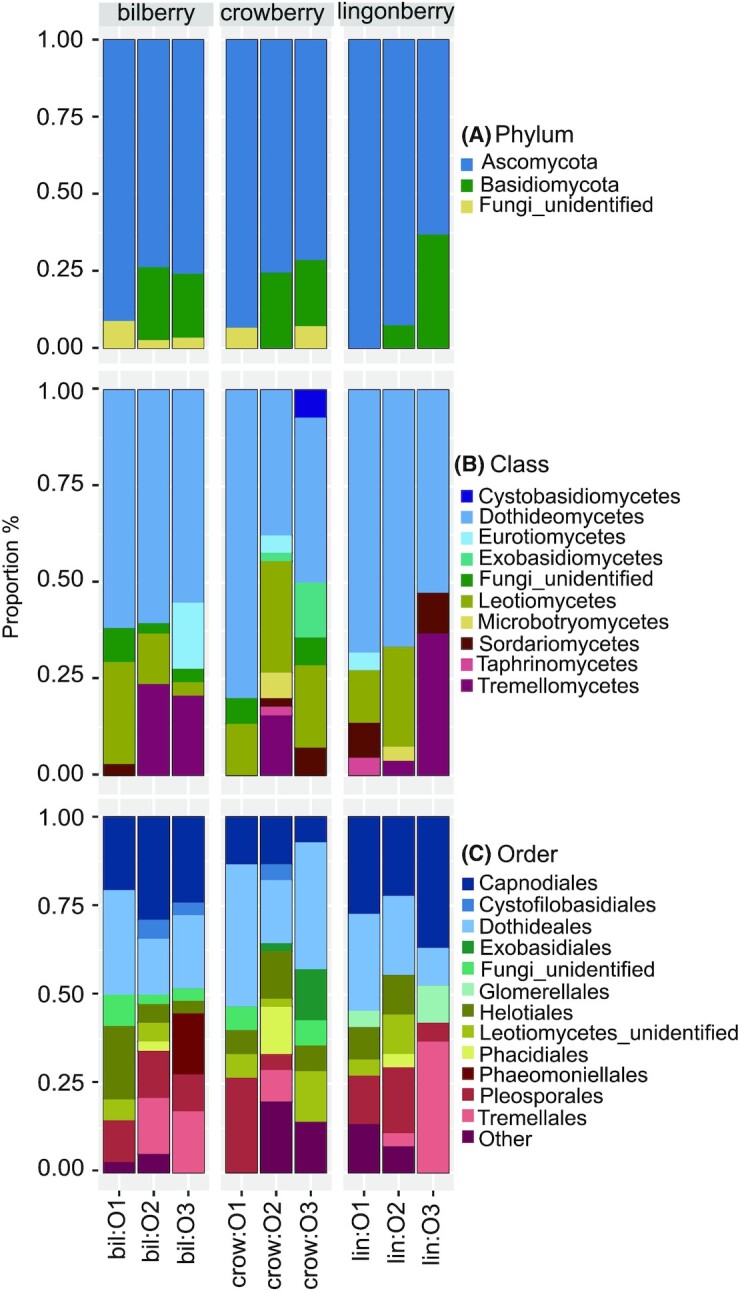
The taxonomic proportions of endophytic fungi at phylum **(A)**, class **(B)** and order **(C)** level generated by R package *phyloseq* v.1.30.0 and *ggplot2* v.3.3.0. Sample names contain the berry species (bil – bilberry, crow – crowberry and lin – lingonberry) and the growth site indicating where the samples were collected (O1, O2 and O3). Other: the combination of all orders having the average proportion across all samples smaller than 1% (*Diaporthales*, *Microbotryomycetes_ord_Incertae_sedis*, *Eurotiales*, *Botryosphaeriales*, *Filobasidiales*, *Sporidiobolales*, *Xylariales*, *Taphrinales*, *Venturiales*, *Sordariales*, *Cyphobasidiales* and *Tremellomycetes* unidentified order).

Of all screened fungal taxa, an average proportion of 82% belonged to *Ascomycota*, followed by 15% *Basidiomycota* and 3% unidentified fungi (Fig. 1A, Appendix S3: Table S3, Supporting Information). At class level, the major class was *Dothideomycetes* (58%), followed by *Leotiomycetes* (16%) and *Tremellomycetes* (11%), while proportions of the remaining fungal classes were less than 5% for each (Fig. 1B, Appendix S3: Table S3, Supporting Information). At order level, the predominant orders were *Dothideales* and *Capnodiales* (24% and 22%, respectively), followed by *Pleosporales*, *Tremellales* and *Helotiales* (12%, 9% and 8.5%, respectively; Fig. 1C, Appendix S3: Table S3, Supporting Information). At the genus level, *Sydowia*, *Cladosporium* and *Vishniacozyma* were the predominant fungal genera (17%, 15% and 8%, respectively; Appendix S3: Table S3, Supporting Information).

All examined bacterial taxa belonged to *Gammaproteobacteria* class (*Proteobacteria* phylum, Appendix S4: Table S4, Supporting Information). The numbers of bacterial orders were limited to *Pseudomonadales*, *Enterobacterales*, *Burkhoderiales* and *Xanthomonadales* (Appendix S4: Table S4, Supporting Information). Due to the low number of bacterial taxa and the absence of bacteria in the lingonberry at site O3, bacteria were not used in further diversity analysis.

When comparing taxonomy profiles between the berry species, the greatest differences were observed between crowberry and the other two berry species. Bilberry and lingonberry had higher proportion of the fungal order *Capnodiales* compared to crowberry (average 25% and 29% vs. 11%, respectively), while *Dothideales* was predominant in crowberry (31% on average; Fig. 1C). *Exobasidiales* fungi (genus *Exobasidium*) were only found in crowberry (sites O2 and O3; Fig. 1C). The bacterial order *Pseudomonadales* (genus *Pseudomonas*) was found in all crowberry samples and bilberry sample at site O2, but not in the rest of the samples (Appendix S4: Table S4, Supporting Information). The fungal genus *Botrytis* was found in all crowberry samples (average 5%) and lingonberry from site O1 (4.5%), but not in the other locations or in bilberry species (Appendix S3: Table S3, Supporting Information).

When comparing the growth sites, the fungal phylum *Basidiomycota* was present in the samples of site O2 and O3 but not site O1 (Fig. 1A). The fungal order *Phacidiales* was found only in samples collected from site O2 (Fig. 1C).

### Diversity and composition of endophytic communities

**Figure 2.**
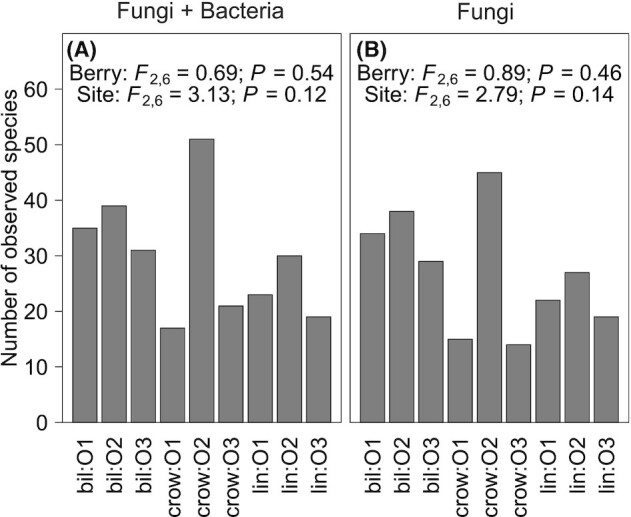
Taxonomic richness of all taxa including both fungi and bacteria **(A)** and only fungal taxa **(B)**. The statistical differences of the taxonomic richness between berry species (Berry) or growth sites (Site) were accessed by one-way ANOVA tests; *F*_degree of freedom_ and *P* values of the tests are presented. Sample names contain the berry species (bil – bilberry, crow – crowberry and lin – lingonberry) and the growth site where the samples were collected (O1, O2 and O3).

Taxonomic richness of the endophytes (including both fungi and bacteria) did not differ among three berry species (ANOVA: *F*_2,6_ = 0.69, *P* > 0.5) or growth sites (ANOVA: *F*_2,6_ = 3.13, *P* > 0.1; Fig. 2A). Similar results were observed when we considered the taxonomy richness of fungi separately (Fig. 2B).

**Figure 3.**
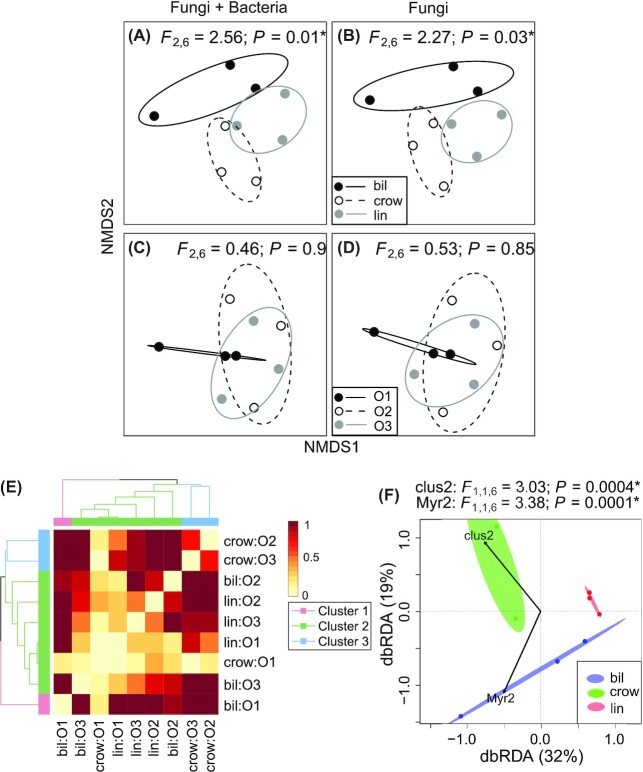
Nonmetric multidimensional scaling (NMDS) ordinations of endophytic community compositions grouped by berry species **(A and B)**, growth sites **(C and D)**, an UPGMA tree **(E)** representing the similarity of the endophytic diversity of the samples, and a dbRDA plot **(F)** visualizing the most important collinearity-removal phenolic variables explaining the endophytic community assemblages. (A–D and F) Ellipses denote 95% confidence intervals around the group centroid based on standard errors. (*) indicates the significance in the statistical tests. Abbreviations: bil – bilberry, crow – crowberry, lin – lingonberry and growth sites (O1, O2 and O3).

Endophytic composition including both fungi and bacteria using Raup–Crick dissimilarity metric differed among the three berry species (PERMANOVA, global test: *F*_2,6_ = 2.56, *P* = 0.01; Fig. 3A, Table 1) but not among the growth sites (PERMANOVA, global test: *F*_2,6_ = 0.46, *P* > 0.5, Fig. 3C, Table 1). Similarly, fungal community composition was different among berry species (*F*_2,6_ = 2.27, *P* = 0.032; Fig. 3B, Table 1) but not among growth sites (*F*_2,6_ = 0.53, *P* > 0.5, Fig. 3D, Table 1). The results were confirmed also with Sørensen dissimilarity index (Appendix S1: Figure S5, Supporting Information and Table 1).

Similarity of endophytic composition of the samples was examined using hierarchical cluster analysis (HCA), where three clusters were identified (Fig. 3E). Crowberry at site O2 and O3 formed one cluster (cluster 3), while bilberry at site O1 formed own cluster (cluster 1) and was separated from the rest of the samples. The second cluster contained the rest of the samples (bilberry at site O2 and O3, crowberry at site O1 and lingonberry from all three sites), in which samples belonged to the same site were more similar to each other and community composition of sites O1 and O3 was more similar compared to that of site O2.

**Figure 4.**
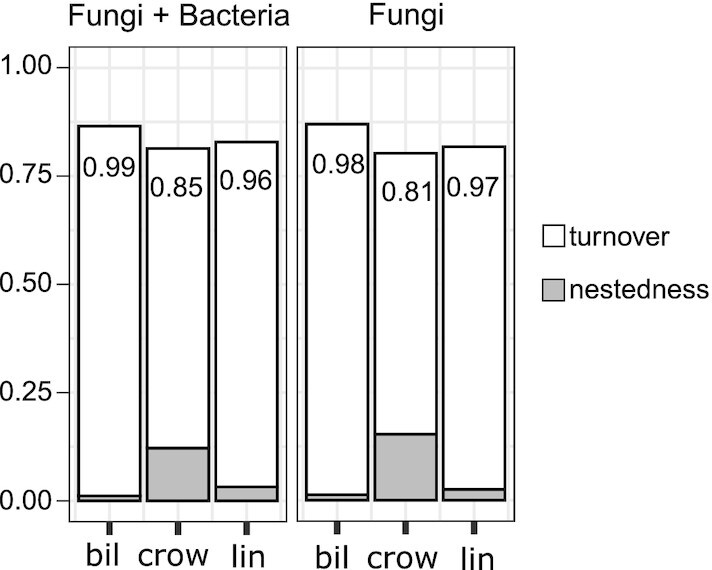
The multiple-site Sørensen dissimilarity (β_SOR_) and its components of turnover (β_SIM_) and nestedness (β_SNE_) of endophytic communities on three berry species. β_ratio_ is the ratio of β_SIM_ to β_SOR_. β_ratio_ < 0.5 indicates that β diversity is driven primarily by nestedness, while β_ratio_ > 0.5 indicates the predominant role of turnover.

When examining the relative importance of turnover and nestedness in determining changes in community composition, we found that turnover component composed the largest fraction of overall dissimilarity (β_ratio_ > 0.8 in all groups, Fig. 4, Appendix S5: Table S5, Supporting Information). The large β_ratio_ values indicated that variation of community composition was primarily related to turnover.

We identified one indicator species for crowberry (*P* < 0.04), *Botrytis cinerea* (species E5). In our dataset, *B. cinerea* was found only in crowberry but not in other berry species.

### Importance of the host phenolic compounds on endophytic structure

A total of 56 compounds were quantified in every berry type (Appendix S5: Table S6, Supporting Information). The most abundant compounds were anthocyanins (ACNs) in bilberry and crowberry (approximately 80% of the total soluble phenolics), while proanthocyanidins (PAs) were the most abundant group of compounds in lingonberry (Appendix S5: Table S7, Supporting Information). The phenolic composition was grouped distinctly according to berry species but not based on growth sites in the ordination space due to the strong difference between the berry species and the close distance between the sites (Appendix S1: Figure S6B, Supporting Information). Comparison of the eigenvalues of principal component axes to the broken stick model revealed that the first two axes explained the most variation in the phenolic data (90% of total variance in the phenolic data). Phenolic composition differed among the berry species (PERMANOVA global test: *F*_2,6_ = 25.9, *P* = 0.004) but not among growth sites (*F*_2,6_ = 0.1, *P* > 0.5).

Pairwise correlation coefficient revealed that the phenolic compound variables were highly collinear (Appendix S1: Figure S6A, Supporting Information); therefore, a new matrix of phenolic compound data was produced to reduce collinearity (Appendix S5: Table S8, Supporting Information). After collinearity reduction, the phenolic compound data were still distinctly different among the three berry species (PERMANOVA global test: *F*_2,6_ = 10.9, *P* = 0.006; Appendix S1: Figure S6C, Supporting Information) but not among the growth sites (PERMANOVA global test: *F*_2,6_ = 0.28, *P* > 0.5).

Model selection for βrc identified two out of eight collinearity-removal phenolic variables significantly affecting the distance-based redundancy analysis (dbRDA) model. They were Clus2 (permutation tests: *F*_1,1,6_ = 3.03, *P* < 0.001) and Myricetin derivative 2 (Myr2; permutation tests: *F*_1,1,6_ = 3.38, *P* = 0.0001; Fig. 3F).

## DISCUSSION

Endophytic communities of wild plant species and reproductive organs have rarely been studied. The present study focused on endophytic communities in the fruits of the most common wild berries in Northern Europe, bilberry, lingonberry and crowberry. We found that (i) the host plant species shape the unique endophytic composition; and (ii) the host phenolic compounds likely influence the endophytic composition.

We used a culture-dependent method to investigate the bacterial and fungal endophyte communities of berry fruits. Although high-throughput sequencing technology is currently popular, it does not produce microbial strains for further investigations. We sampled the fruits of the three berry species growing in mixed populations to evaluate effect of growth site in defining the community composition. We focused on both bacterial and fungal endophytes, while most of the previous endophyte studies have focused on either of them (Dugan, Lupien and Grove 2002; Compant *et al*. 2011; Tadych *et al*. 2012; Glassner *et al*. 2015).

Fungi were predominant in the total isolated endophytic taxa in the present study (172 taxa) while bacteria only composed a small proportion (18 taxa). A low number of endophytic bacteria isolated from plant reproductive organs is agreeable with Compant *et al*. (2011) and Glassner *et al*. (2015). However, due to the low quantity of bacterial taxa and the absence of bacteria in one of our sample (lingonberry at site O3), we did not include bacteria as an individual group in the diversity analysis.

### Host species shapes the community composition of endophytes

Our results indicated that host plant species deeply influences the overall endophytic composition of wild berry fruits. When examining fungal community composition separately, we found that fungal communities were specifically affected by berry species. This dependency of fungi was likely because fungi made up 91% of the total endophytic taxa in the present study. Host species selecting their microbiomes from the same reservoir of microbes has earlier been studied for grapevines and weeds located in the same vineyard (Samad *et al*. 2017), and only seven bacterial taxa (12.3%) were shared in all plants and compartments studied.

Community composition were driven by turnover component, supporting the idea that endophytic communities were host specific rather than nested subsets of high-diversity communities. We found one indicator species for crowberry fruits, *B. cinerea*. Although *B. cinerea* strains are commonly found as pathogens, there are strains that live inside plant tissue without causing symptoms of disease (van Kan, Shaw and Grant-Downton 2014). We did not find indicator species for bilberry or lingonberry fruits, which might be due to the small sample size. However, the low degree of overlapping of endophytic communities between the three berry species in each location reflects the unique endophytic communities selected by the host species. Specifically, the shared endophytes were *Pleosporales* sp. E149 for location O1, *Cladosporium* sp. E13 and *Pleosporales* sp. E150 for location O2, and *Sydowia* sp. E77 for location O3.

### The host phenolic compounds can affect endophytic community composition

We observed a potential impact of specific phenolic variables on shaping the endophytic communities by the dbRDA analysis. The collinearity-removal phenolic variable clus2 (which was the cluster score of many original phenolic compound variables after reducing collinearity) and Myr2 (Myricetin derivative 2) strongly corresponded with these features of the community structure, which suggested that they were likely involved in the metabolic crosstalk between the host and endophytes in these samples. Specifically, clus2 appeared to separate the endophytic community of crowberry from those of bilberry and lingonberry. On the other hand, Myr2 – which was only detected in the bilberry samples – might be one of the factors that separates the community of endophytes in bilberry from those in crowberry and lingonberry.

The interaction between the host plant and its symbionts takes place through their metabolic crosstalk (Lòpez-Fernàndez *et al*. 2016). Endophytes can alter the host metabolism, and the host responds to the endophytic colonization by shifting its metabolic profile to favor colonization by the symbionts. Although phenolic compounds are mainly stored in the plant vacuole, they can be imported and exported by specific transporters (De Brito Francisco and Martinoia 2018). Accumulation of specific phenolic compounds in response to interaction with either a pathogen or an endophyte has been reported in bilberry leaves (Koskimäki *et al*. 2009). Therefore, the variations in phenolic compound composition between berry species, observed in our study, could play a role in the crosstalk between the host and endophytes. However, a more thorough research is required to confirm our preliminary result.

### Unknown environmental factors partly shape the endophyte communities

The samples at the growth site O2 tended to have a higher number of species compared to those at the other sites. Site O2 is boreal forest with high density of trees and diverse plant species such as birches (*Betula pendula* Roth and *B. pubescens* Ehrh.), pine (*Pinus sylvestris* L.), other wild berries (*V. uliginosum* L., ) and grasses. The location is close to a lake with no built environment in the vicinity, and it is less disturbed by urban activities (Appendix S1: Figure S1, Supporting Information and Appendix S2: Table S1, Supporting Information). The locations O1 and O3 have lower density of trees and are less diverse with respect to plant species compared to the location O2. Since these two locations are surrounded by buildings and roads, they likely are more affected by urban activities (Appendix S1: Figure S1, Supporting Information and Appendix S2: Table S1, Supporting Information). As the endophytes in the above-ground plant tissues are derived from the phyllosphere or from the seeds (Compant *et al*. 2011; Hardoim *et al*. 2015), we assumed that the fruits at the location O2 with higher diversity and density of species would be colonized by more microbial species.

Our results also indicated that unmeasured environmental factors likely have affected the communities. Ideally, if only host plant species had an effect on endophytic composition, there would be three clusters grouped by each berry species in the dendrogram. However, there was a mixed effect of berry species and unknown factors influencing the grouping, in particular in cluster 2, which contained the samples from all three berry species. In this cluster, the samples of site O1 and site O3 were more similar compared to site O2. Similar patterns of mixed effects of locations and host species have been reported by Sun *et al*., (2020). Based on the growth sites and related literature, we assumed that environmental factors, such as urbanization might also influence endophytic community structure. Urbanization can cause microclimate changes, such as heat-island effects and chemical pollution, forest fragmentation and isolation (Matsumura and Fukuda 2013). Similar to the wild berries at sites O1 and O3, tree species studied in the urban areas generally share more similar endophytic communities compared to those in forests (Matsumura and Fukuda 2013).

### The environment is a likely source of endophytes

The fact that endophytic communities of wild berries varied between growth sites suggests that they originated from the environment. The degree of overlap in endophytic communities between the sites of each host was low, which indicates that the majority of endophytes originated from the environment rather than being seed-borne. Specifically, the shared species were *Sydowia* sp. E71 for bilberry, *B. cinerea* E76 and *Sydowia* sp. E77 for crowberry and *Cladosporium* sp. E13 and *Sydowia* sp. E77 for lingonberry.

### Fruits of wild berries are a favored organ by fungal endophytes

High fungal diversity and low number of bacteria (91% and 9%, respectively) might be due to various fungal strategies of colonizing the host plant (Carroll 1988; Stone, Polishook and White 2004; Hardoim *et al*. 2015), while the majority of bacterial endophytes originate from the rhizosphere (Compant, Clément and Sessitsch 2010; Philippot *et al*. 2013; Hardoim *et al*. 2015). Specifically, horizontally transmitted shoot endophytic fungi can enter the plant by air-borne spores via air and water (Rodriguez *et al*. 2009). On the other hand, the soil-derived endophytic bacteria need to travel from roots to above-ground plant parts (Hardoim *et al*. 2015). Moreover, the ability of fungi to utilize more readily sugars might be a factor favoring high fungal populations in fruits (Souza Guimaraes 2012). Among the four classes of fungal endophytes, representatives of classes 1, 2 and 3 generally colonize the above-ground plant parts. Apart from the specific group of Clavicipitacean endophytes of class 1, fungal endophytes of classes 2 and 3 have a broad host range (Rodriguez *et al*. 2009). Because both growth sites and plant species shaped endophytic communities in the wild berry fruits, the fungi likely belong to classes 2 and 3 of fungal endophytes.

### New endophytic taxa were found in our study

When comparing our data with published reports of endophytes in reproductive organs, we found commonly detected fungi and bacteria. One bacterial genus, *Pseudomonas*, has been found in grapes, cucurbits and strawberry (Kukkurainen *et al*. 2005; Compant *et al*. 2011; Glassner *et al*. 2015). Many fungal genera identified in this study have been detected in several plant species, such as *Cladosporium* in coffee berries, grass seeds, grape berries and cranberry ovary (Dugan, Lupien and Grove 2002; Dugan and Lupien 2003; Vega *et al*. 2008; Tadych *et al*. 2012). *Alternaria* has been detected in grape berries, cranberry ovaries and grass seeds (Dugan, Lupien and Grove 2002; Dugan and Lupien 2003; Tadych *et al*. 2012). *Penicillium* and *Phyllosticta* have also been found in cranberry ovaries (Tadych *et al*. 2012). *Rhodosporidiobolus colostri* has been detected in *Malus domestica* and *Pyrus communis* fruits (Glushakova and Kachalkin 2017), while *Aureobasidium pullulans* was found in cherries and bean seeds (Schena *et al*. 2003; Parsa *et al*. 2016). Lastly, *B. cinerea* has been identified in grape berries and cranberry ovary (Dugan, Lupien and Grove 2002; Tadych *et al*. 2012).

We also found unique genera and species, which have not previously been reported as endophytes. These taxa belonged to *Angustimassarina*, *Dothidea*, *Fellozyma*, *Pseudohyphozyma*, *Hannaella coprosmae* and *Oberwinklerozyma straminea*. In general, members of the genus *Dothidea* are rarely reported. A species belonging to *Angustimassarina* is associated with other fungi, growing within ascomata of *Ascomycetes* (Hyde *et al*. 2017). *Hannaella coprosmae*, a basidiomycetous yeast, has been found on the phylloplane of plant leaves (Li *et al*. 2020), and *Oberwinklerozyma straminea* (or *Rhodotorula straminea*) is a yeast found in dead needle litter of *Picea abies* L. and *Pinus sylvestris* L. (Golubev and Scorzetti 2010).

## CONCLUSIONS

We discovered a clear effect of host plant species on shaping the endophytic community composition. Our data suggests that the majority of berry fruit-associated endophytes originate from the environment and are selected by the host species. The phenolic compounds of the host can play an important role in the metabolic crosstalk and colonization by the endophytes. The endophytic community structures differed between the berry species, and each berry species harbored a unique endophytic community of microbes. We also found one specific indicator species for crowberry fruits,  *B. cinerea*,  i.e. the endophytic strain living inside plant tissue without causing symptoms of disease.  Moreover, we found that wild berry fruits hosted mainly fungal endophytes over bacterial ones, which might be due to the differences in the colonization strategies of fungi and bacteria. The low degree of overlapping of endophytic communities between the three species in each location reflects the unique endophytic communities selected by the host. Thus, our data may open the door for authentication analyses of wild berry species using endophytic communities.

## Supplementary Material

fiab097_Supplement_File

## References

[bib1] Alvin A , Miller KI, Neilan BA. Exploring the potential of endophytes from medicinal plants as sources of antimycobacterial compounds. Microbiol Res. 2014;169:483–95. 24582778 10.1016/j.micres.2013.12.009 PMC7126926

[bib2] Anderson MJ , Crist TO, Chase JM et al. Navigating the multiple meanings of β diversity: a roadmap for the practicing ecologist. Ecol Lett. 2011;14:19–28. 21070562 10.1111/j.1461-0248.2010.01552.x

[bib3] Baselga A , Orme CDL. Betapart: an R package for the study of beta diversity. Methods Ecol Evol. 2012;3:808–12.

[bib4] Baselga A , Orme D, Villeger S et al. Partitioning beta diversity into turnover and nestedness components. Cran. 2018;19:134–43.

[bib5] Bodenhausen N , Horton MW, Bergelson J. Bacterial communities associated with the leaves and the roots of *Arabidopsis thaliana*. PLoS ONE. 2013;8:e56329. 23457551 10.1371/journal.pone.0056329 PMC3574144

[bib8] Carroll G . Fungal endophytes in stems and leaves: from latent pathogen to mutualistic symbiont. Ecology. 1988;69:2–9.

[bib9] Chase JM , Kraft NJB, Smith KG et al. Using null models to disentangle variation in community dissimilarity from variation in α-diversity. Ecosphere. 2011;2:art24–11.

[bib10] Chou H-H , Holmes MH. DNA sequence quality trimming and vector removal. Bioinformatics. 2001;17:1093–104. 11751217 10.1093/bioinformatics/17.12.1093

[bib11] Compant S , Clément C, Sessitsch A. Plant growth-promoting bacteria in the rhizo- and endosphere of plants: their role, colonization, mechanisms involved and prospects for utilization. Soil Biol Biochem. 2010;42:669–78.

[bib12] Compant S , Mitter B, Colli-Mull JG et al. Endophytes of grapevine flowers, berries, and seeds: identification of cultivable bacteria, comparison with other plant parts, and visualization of niches of colonization. Microb Ecol. 2011;62:188–97. 21625971 10.1007/s00248-011-9883-y

[bib6] De Brito Francisco R , Martinoia E. The vacuolar transportome of plant specialized metabolites. Plant Cell Physiol. 2018;59:1326–36. 29452376 10.1093/pcp/pcy039

[bib7] De Cáceres M , Legendre P. Associations between species and groups of sites: Indices and statistical inference. Ecology. 2009. DOI:10.1890/08-1823.1. 20120823

[bib13] Denisov GA , Arehart AB, Curtin MD. US6681186B1 - System and method for improving the accuracy of DNA sequencing and error probability estimation through application of a mathematical model to the analysis of electropherograms. Google Patents, 2004.

[bib14] Dormann CF , Elith J, Bacher S et al. Collinearity: a review of methods to deal with it and a simulation study evaluating their performance. Ecography. 2013;36:27–46.

[bib16] Dugan FM , Lupien SL, Grove GG. Incidence, aggressiveness and *in planta* interactions of *Botrytis cinerea* and other filamentous fungi quiescent in grape berries and dormant buds in central Washington State. J Phytopathol. 2002;150:375–81.

[bib15] Dugan FM , Lupien SL. Filamentous fungi quiescent in seeds and culm nodes of weedy and forage grass species endemic to the Palouse Region of Washington and Idaho. Mycopathologia. 2003;156:31–40. 10.1023/a:1021399301542 12715945

[bib17] Eevers N , Gielen M, Sánchez-López A et al. Optimization of isolation and cultivation of bacterial endophytes through addition of plant extract to nutrient media. Microb Biotechnol. 2015;8:707–15. 25997013 10.1111/1751-7915.12291 PMC4476825

[bib18] Ek S , Kartimo H, Mattila S et al. Characterization of phenolic compounds from lingonberry (*Vaccinium vitis-idaea*). J Agric Food Chem. 2006;54:9834–42. 17177509 10.1021/jf0623687

[bib19] Fisher PJ , Anson AE, Petrini O. Novel antibiotic activity of an endophytic *Cryptosporiopsis* sp. isolated from *Vaccinium myrtillus*. Trans Br Mycol Soc. 1984;83:145–8.

[bib20] Fox J , Weisberg S. CAR - An R Companion to Applied Regression. Sage, Thousand Oaks, CA. 2019. https://socialsciences.mcmaster.ca/jfox/Books/Companion/.

[bib21] Ganguly T , Chen P, Teetsel R et al. High-throughput sequencing of high copy number plasmids from bacterial cultures by heat lysis. BioTechniques. 2005;39:304–8. 16206901 10.2144/05393BM02

[bib22] Gdanetz K , Trail F. The wheat microbiome under four management strategies, and potential for endophytes in disease protection. Phytobiomes J. 2017;1:158–68.

[bib23] Glassner H , Zchori-Fein E, Compant S et al. Characterization of endophytic bacteria from cucurbit fruits with potential benefits to agriculture in melons (*Cucumis melo* L.). FEMS Microbiol Ecol. 2015;91:fiv074. 26183916 10.1093/femsec/fiv074

[bib24] Glushakova AM , Kachalkin A V. Endophytic yeasts in *Malus domestica* and *Pyrus communis* fruits under anthropogenic impact. Microbiology. 2017;86:128–35. 30207434

[bib25] Golubev WI , Scorzetti G. *Rhodotorula rosulata* sp. nov., *Rhodotorula silvestris* sp. nov. and *Rhodotorula straminea* sp. nov., novel *myo*-inositol-assimilating yeast species in the Microbotryomycetes. Int J Syst Evol Microbiol. 2010;60:2501–6. 19915106 10.1099/ijs.0.016303-0

[bib26] Häkkinen S , Heinonen M, Kärenlampi S et al. Screening of selected flavonoids and phenolic acids in 19 berries. Food Res Int. 1999;32:345–53.

[bib27] Hardoim PR , Hardoim CCP, van Overbeek LS et al. Dynamics of seed-borne rice endophytes on early plant growth stages. PLoS ONE. 2012;7:e30438. 22363438 10.1371/journal.pone.0030438 PMC3281832

[bib28] Hardoim PR , van Overbeek LS, Berg G et al. The hidden world within plants: ecological and evolutionary considerations for defining functioning of microbial endophytes. Microbiol Mol Biol Rev. 2015;79:293–320. 26136581 10.1128/MMBR.00050-14 PMC4488371

[bib29] Heinonen M . Antioxidant activity and antimicrobial effect of berry phenolics - A Finnish perspective. Mol Nutr Food Res. 2007;51:684–91. 17492800 10.1002/mnfr.200700006

[bib30] Hodgson S , de Cates C, Hodgson J et al. Vertical transmission of fungal endophytes is widespread in forbs. Ecol Evol. 2014;4:1199–208. 24834319 10.1002/ece3.953 PMC4020682

[bib31] Hyde KD , Norphanphoun C, Abreu VP et al. Fungal diversity notes 603–708: taxonomic and phylogenetic notes on genera and species. Fungal Divers. 2017;87:1–235.

[bib32] Ihrmark K , Bödeker ITM, Cruz-Martinez K et al. New primers to amplify the fungal ITS2 region - evaluation by 454-sequencing of artificial and natural communities. FEMS Microbiol Ecol. 2012;82:666–77. 22738186 10.1111/j.1574-6941.2012.01437.x

[bib33] Jaakola L , Pirttilä AM, Vuosku J et al. Method based on electrophoresis and gel extraction for obtaining genomic DNA-free cDNA without DNase treatment. BioTechniques. 2004;37:744–8. 15560129 10.2144/04375BM06

[bib34] Jackson DA . Stopping rules in principal components analysis: a comparison of heuristical and statistical approaches. Ecology. 1993;74:2204–14.

[bib36] Koskimäki JJ , Hokkanen J, Jaakola L et al. Flavonoid biosynthesis and degradation play a role in early defence responses of bilberry (*Vaccinium myrtillus*) against biotic stress. Eur J Plant Pathol. 2009;125:629–40.

[bib37] Krishnan P , Bhat R, Kush A et al. Isolation and functional characterization of bacterial endophytes from *Carica papaya* fruits. J Appl Microbiol. 2012;113:308–17. 22587617 10.1111/j.1365-2672.2012.05340.x

[bib38] Kukkurainen S , Leino A, Vähämiko S et al. Occurrence and location of endophytic bacteria in garden and wild strawberry. HortScience. 2005;40:348–52.

[bib39] Li AH , Yuan FX, Groenewald M et al. Diversity and phylogeny of basidiomycetous yeasts from plant leaves and soil: proposal of two new orders, three new families, eight new genera and one hundred and seven new species. Stud Mycol. 2020;96:17–140. 32206137 10.1016/j.simyco.2020.01.002 PMC7082220

[bib40] Llorens E , Sharon O, Camañes G et al. Endophytes from wild cereals protect wheat plants from drought by alteration of physiological responses of the plants to water stress. Environ Microbiol. 2019;21:3299–312. 10.1111/1462-2920.14530 30637909

[bib41] Lòpez-Fernàndez S , Compant S, Vrhovsek U et al. Grapevine colonization by endophytic bacteria shifts secondary metabolism and suggests activation of defense pathways. Plant Soil. 2016;405:155–75.

[bib42] Lòpez-Fernàndez S , Mazzoni V, Pedrazzoli F et al. A phloem-feeding insect transfers bacterial endophytic communities between grapevine plants. Front Microbiol. 2017;8:834. 28555131 10.3389/fmicb.2017.00834 PMC5430944

[bib43] Lugtenberg BJJ , Caradus JR, Johnson LJ. Fungal endophytes for sustainable crop production. FEMS Microbiol Ecol. 2016;92:194. 10.1093/femsec/fiw194 27624083

[bib44] Määttä-Riihinen KR , Kähkönen MP, Törrönen AR et al. Catechins and procyanidins in berries of *Vaccinium* species and their antioxidant activity. J Agric Food Chem. 2005;53:8485–91. 16248542 10.1021/jf050408l

[bib45] Manganaris GA , Goulas V, Vicente AR et al. Berry antioxidants: small fruits providing large benefits. J Sci Food Agric. 2014;94:825–33. 24122646 10.1002/jsfa.6432

[bib46] Marquez LM , Redman RS, Rodriguez RJ et al. A virus in a fungus in a plant: three-way symbiosis required for thermal tolerance. Science. 2007;315:513–5. 17255511 10.1126/science.1136237

[bib47] Matsumura E , Fukuda K. A comparison of fungal endophytic community diversity in tree leaves of rural and urban temperate forests of Kanto district, eastern Japan. Fungal Biol. 2013;117:191–201. 23537876 10.1016/j.funbio.2013.01.007

[bib48] Miina J , Hotanen JP, Salo K. Modelling the abundance and temporal variation in the production of bilberry (*Vaccinium myrtillus* L.) in Finnish mineral soil forests. Silva Fenn. 2009;43:577–93.

[bib49] Nilsson RH , Larsson KH, Taylor AFS et al. The UNITE database for molecular identification of fungi: handling dark taxa and parallel taxonomic classifications. Nucleic Acids Res. 2019;47:D259–64. 30371820 10.1093/nar/gky1022 PMC6324048

[bib50] Ofek-Lalzar M , Gur Y, Ben-Moshe S et al. Diversity of fungal endophytes in recent and ancient wheat ancestors *Triticum dicoccoides* and *Aegilops sharonensis*. FEMS Microbiol Ecol. 2016;92:152. 10.1093/femsec/fiw152 27402714

[bib51] Oksanen J , F. Guillaume Blanchet RK, Legendre P et al. Package “vegan”. Community ecology package version 2.5-6. R Packag version 340. 2019. https://CRAN.R-project.org/package=vegan.

[bib52] Ottesen AR , González Peña A, White JR et al. Baseline survey of the anatomical microbial ecology of an important food plant: *s**olanum lycopersicum* (tomato). BMC Microbiol. 2013;13:114. 23705801 10.1186/1471-2180-13-114 PMC3680157

[bib53] Pacifico D , Squartini A, Crucitti D et al. The role of the endophytic microbiome in the grapevine response to environmental triggers. Front Plant Sci. 2019;10:1256. 31649712 10.3389/fpls.2019.01256 PMC6794716

[bib54] Pan XX , Yuan MQ, Xiang SY et al. The symbioses of endophytic fungi shaped the metabolic profiles in grape leaves of different varieties. PLoS ONE. 2020;15:e0238734. 32915849 10.1371/journal.pone.0238734 PMC7485881

[bib55] Parsa S , García-Lemos AM, Castillo K et al. Fungal endophytes in germinated seeds of the common bean, *Phaseolus vulgaris*. Fungal Biol. 2016;120:783–90. 27109374 10.1016/j.funbio.2016.01.017 PMC4857701

[bib56] Philippot L , Raaijmakers JM, Lemanceau P et al. Going back to the roots: the microbial ecology of the rhizosphere. Nat Rev Microbiol. 2013;11:789–99. 24056930 10.1038/nrmicro3109

[bib57] Puri SC , Nazir A, Chawla R et al. The endophytic fungus *Trametes hirsuta* as a novel alternative source of podophyllotoxin and related aryl tetralin lignans. J Biotechnol. 2006;122:494–510. 16375985 10.1016/j.jbiotec.2005.10.015

[bib58] Quast C , Pruesse E, Yilmaz P et al. The SILVA ribosomal RNA gene database project: improved data processing and web-based tools. Nucleic Acids Res. 2013;41:D590–6. 23193283 10.1093/nar/gks1219 PMC3531112

[bib59] R Core Development Team, R Core Team, R Foundation for Statistical Computing . R: A Language and Environment for Statistical Computing. 2019.

[bib60] Rai M , Rathod D, Agarkar G et al. Fungal growth promotor endophytes: a pragmatic approach towards sustainable food and agriculture. Symbiosis. 2014;62:63–79.

[bib61] Rajala T , Velmala SM, Tuomivirta T et al. Endophyte communities vary in the needles of Norway spruce clones. Fungal Biol. 2013;117:182–90. 23537875 10.1016/j.funbio.2013.01.006

[bib62] Ramette A. Multivariate analyses in microbial ecology. FEMS Microbiol Ecol. 2007;62:142–60. 17892477 10.1111/j.1574-6941.2007.00375.x PMC2121141

[bib63] Rodriguez RJ , White JF, Arnold AE et al. Fungal endophytes: diversity and functional roles: tansley review. New Phytol. 2009;182:314–30. 19236579 10.1111/j.1469-8137.2009.02773.x

[bib64] Samad A , Trognitz F, Compant S et al. Shared and host-specific microbiome diversity and functioning of grapevine and accompanying weed plants. Environ Microbiol. 2017;19:1407–24. 27871147 10.1111/1462-2920.13618

[bib65] Schena L , Nigro F, Pentimone I et al. Control of postharvest rots of sweet cherries and table grapes with endophytic isolates of *Aureobasidium pullulans*. Postharvest Biol Technol. 2003;30:209–20.

[bib66] Shahzad R , Khan AL, Bilal S et al. What is there in seeds? Vertically transmitted endophytic resources for sustainable improvement in plant growth. Front Plant Sci. 2018;9:24. 29410675 10.3389/fpls.2018.00024 PMC5787091

[bib67] Singh DK , Sharma VK, Kumar J et al. Diversity of endophytic mycobiota of tropical tree *Tectona grandis* Linn.f.: spatiotemporal and tissue type effects. Sci RepSci Rep. 2017;7:3745–14. 10.1038/s41598-017-03933-0 PMC5473821 28623306

[bib68] Souza Guimaraes LH. Carbohydrates from biomass: sources and transformation by microbial enzymes. In: Carbohydrates - Comprehensive Studies on Glycobiology and Glycotechnology. InTech, 2012, 441–58.

[bib69] Stone JK , Polishook JD, White JF. Endophytic fungi. In: Biodiversity of Fungi. Elsevier, 2004, 241–70.

[bib70] Subramanian P , Mageswari A, Kim K et al. Psychrotolerant endophytic *Pseudomonas* sp. strains OB155 and OS261 induced chilling resistance in tomato plants (*Solanum lycopersicum* Mill.) by activation of their antioxidant capacity. Mol Plant-Microbe Interact. 2015;28:1073–81. 26075827 10.1094/MPMI-01-15-0021-R

[bib71] Sun X , Kosman E, Sharon O et al. Significant host- and environment-dependent differentiation among highly sporadic fungal endophyte communities in cereal crops-related wild grasses. Environ Microbiol. 2020;22:3357–74. 32483901 10.1111/1462-2920.15107

[bib72] Tadych M , Bergen MS, Johnson-Cicalese J et al. Endophytic and pathogenic fungi of developing cranberry ovaries from flower to mature fruit: diversity and succession. Fungal Divers. 2012;54:101–16.

[bib73] Tejesvi M V. , Picart P, Kajula M et al. Identification of antibacterial peptides from endophytic microbiome. Appl Microbiol Biotechnol. 2016;100:9283–93. 27541748 10.1007/s00253-016-7765-4

[bib74] Tian Q , Giusti MM, Stoner GD et al. Screening for anthocyanins using high-performance liquid chromatography coupled to electrospray ionization tandem mass spectrometry with precursor-ion analysis, product-ion analysis, common-neutral-loss analysis, and selected reaction monitoring. J Chromatogr A. 2005;1091. DOI:10.1016/j.chroma.2005.07.036. 16395794

[bib75] Unterseher M , Gazis R, Chaverri P et al. Endophytic fungi from Peruvian highland and lowland habitats form distinctive and host plant-specific assemblages. Biodivers Conserv. 2013;22:999–1016.

[bib35] van Kan JAL , Shaw MW, Grant-Downton RT. *Botrytis* species: relentless necrotrophic thugs or endophytes gone rogue?. Mol Plant Pathol. 2014;15:957–61. 24754470 10.1111/mpp.12148 PMC6638755

[bib76] Vega FE , Posada F, Catherine Aime M et al. Entomopathogenic fungal endophytes. Biol Control. 2008;46:72–82.

[bib77] Walters W , Hyde ER, Berg-Lyons D et al. Improved bacterial 16S rRNA gene (V4 and V4-5) and fungal internal transcribed spacer marker gene primers for microbial community surveys. mSystems. 2016;1:e00009–15. 10.1128/mSystems.00009-15 PMC5069754 27822518

[bib78] Yang MZ , Ma M Di, Yuan MQ et al. Fungal endophytes as a metabolic fine-tuning regulator for wine grape. PLoS ONE. 2016;11:e0163186. 27656886 10.1371/journal.pone.0163186 PMC5033586

[bib79] Zafra-Stone S , Yasmin T, Bagchi M et al. Berry anthocyanins as novel antioxidants in human health and disease prevention. Mol Nutr Food Res. 2007;51:675–83. 17533652 10.1002/mnfr.200700002

