# Supplementary material for: Host species shape the community structure of culturable endophytes in fruits of wild berry species (Vaccinium myrtillus L., Empetrum nigrum L. and Vaccinium vitis-idaea L.)
Source: FEMS Microbiol Ecol. 2021 Jul 12;97(8):fiab097. doi: 10.1093/femsec/fiab097 (PMC8292141; doi:10.1093/femsec/fiab097)
Supplement: fiab097_Supplement_File [file fiab097_supplement_file.zip › Appendix-S1.docx]

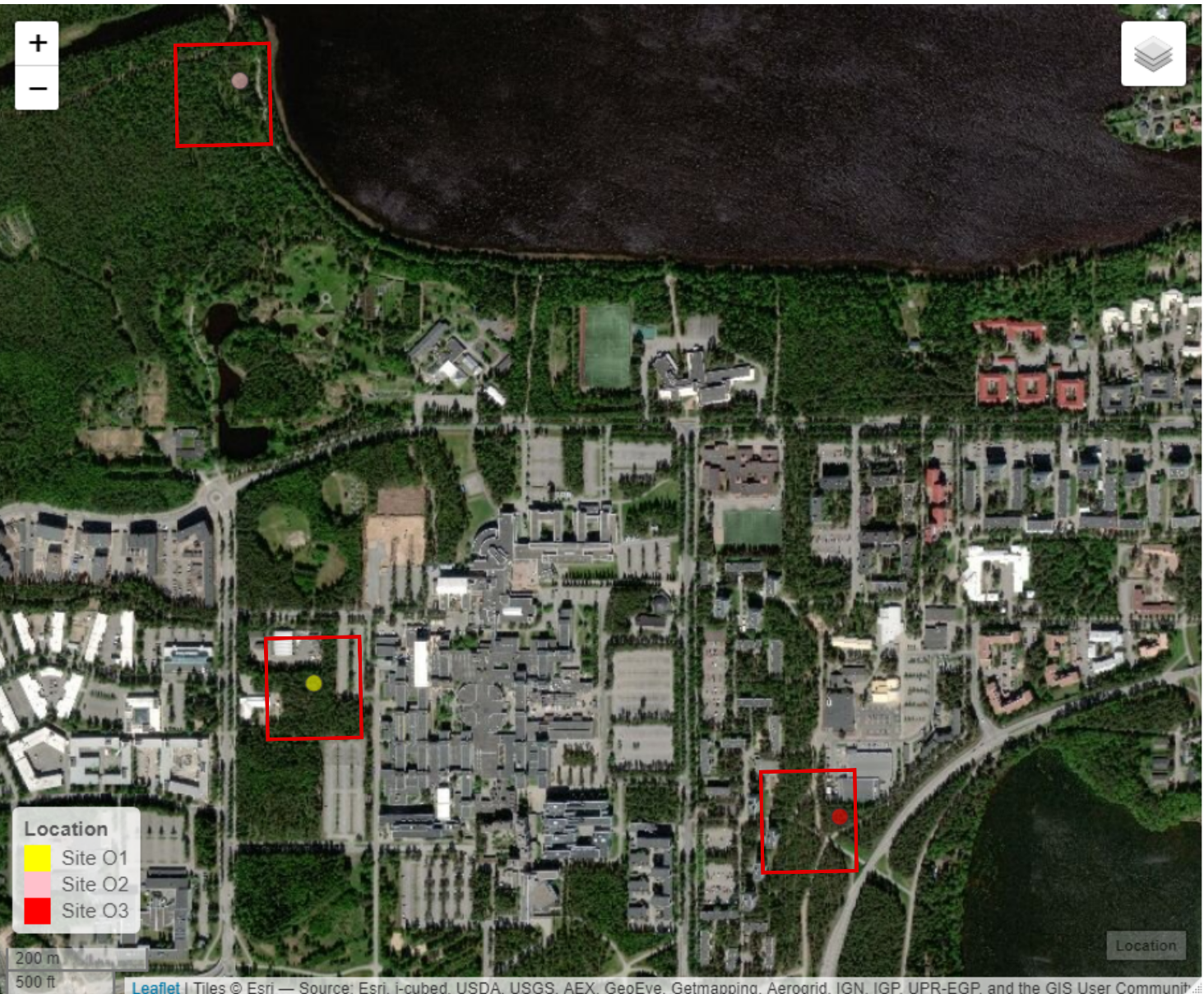


Figure S1: Sampling sites in Oulu, Finland (O1: 65.059611 N 25.461306 E; O2: 65.067111 N 25.459111 E; O3: 65.057944 N 25.476806 E). Each site contains three berry species: bilberry, crowberry, and lingonberry. The distance between sampling sites: O1-O2: 750 m; O1-O3: 400 m; O2-O3: 950 m. Red squares indicate the area where the samples were collected.


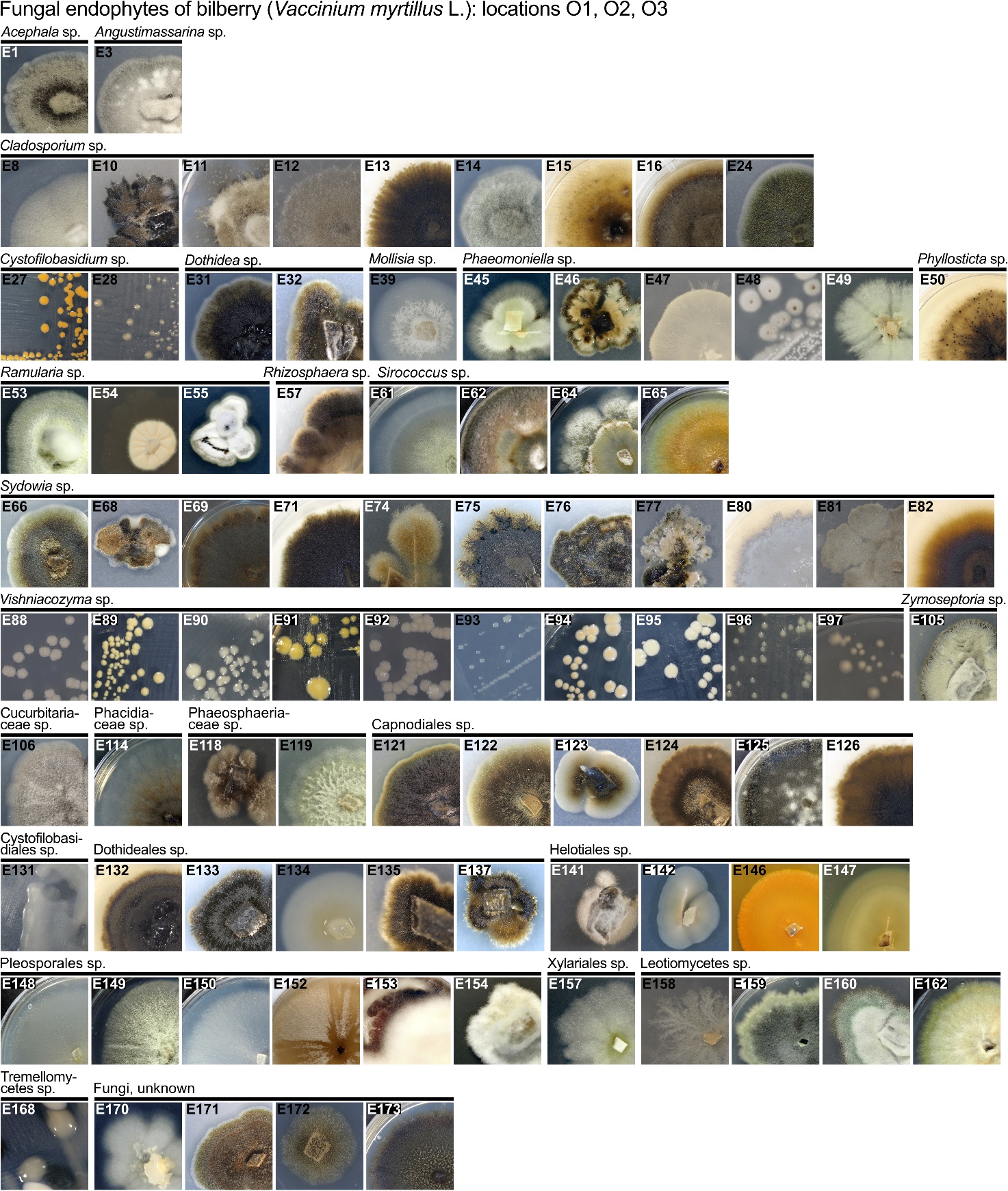


Figure S2: Fungal endophytes of bilberry from three growth sites. The species were marked by their ID (e.g. E1) and grouped by their closest ancestors (e.g. Cladosporium sp.).


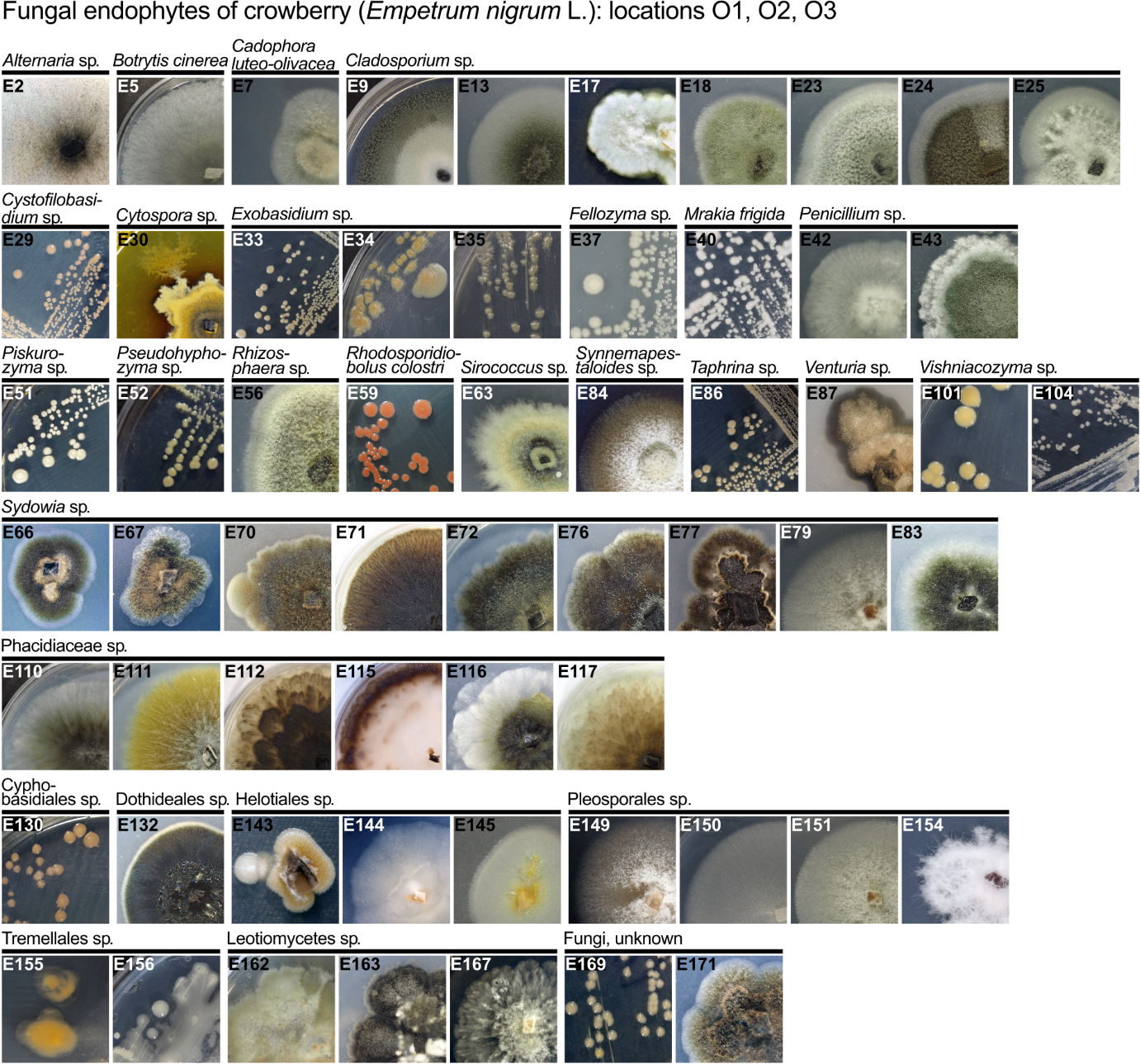


Figure S3: Fungal endophytes of crowberry from three growth sites. The species were marked by their ID (e.g. E1) and grouped by their closest ancestors (e.g. Cladosporium sp.).


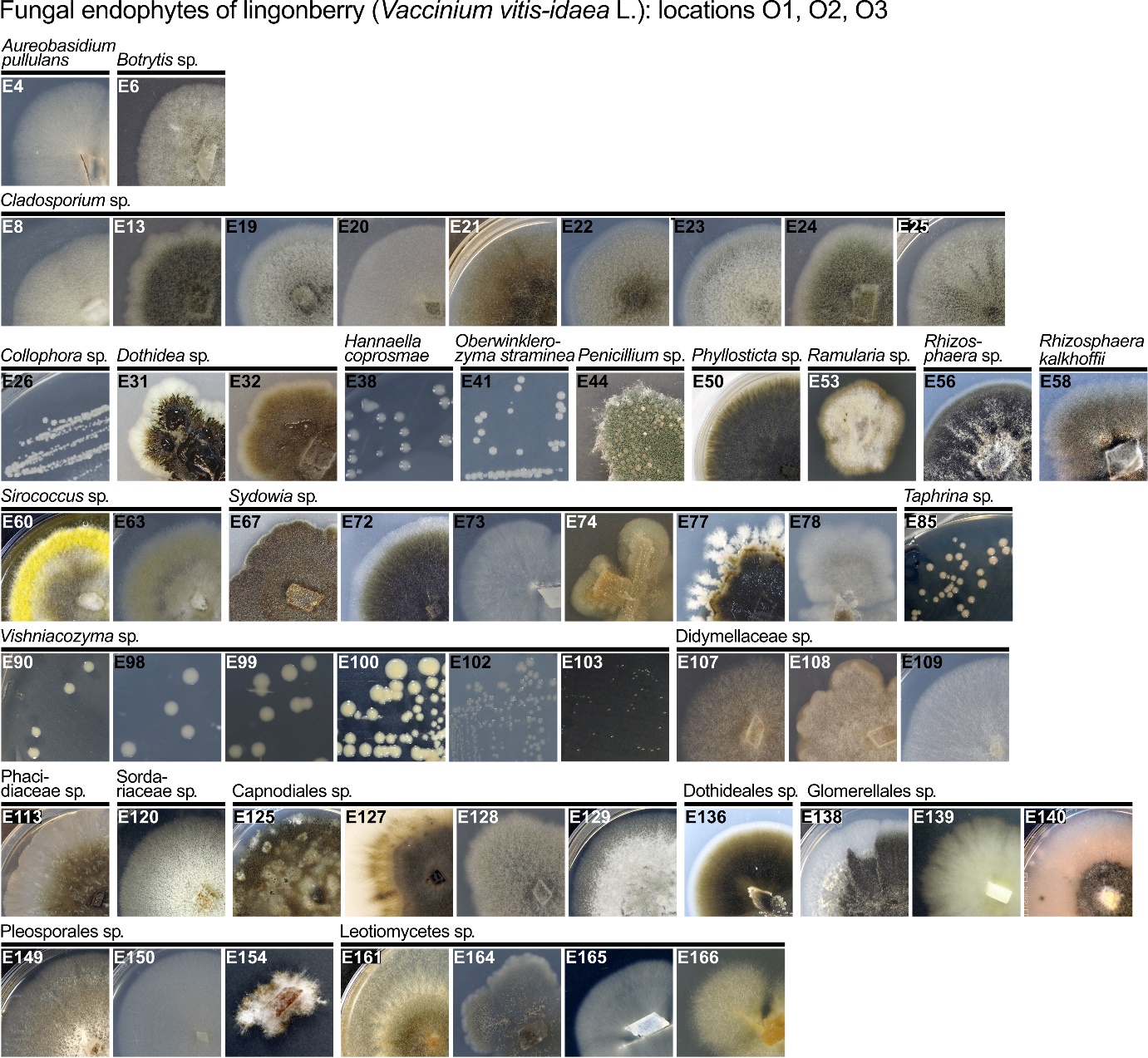


Figure S4: Fungal endophytes of lingonberry from three growth sites. The species were marked by their ID (e.g. E1) and grouped by their closest ancestors (e.g. Cladosporium sp.).


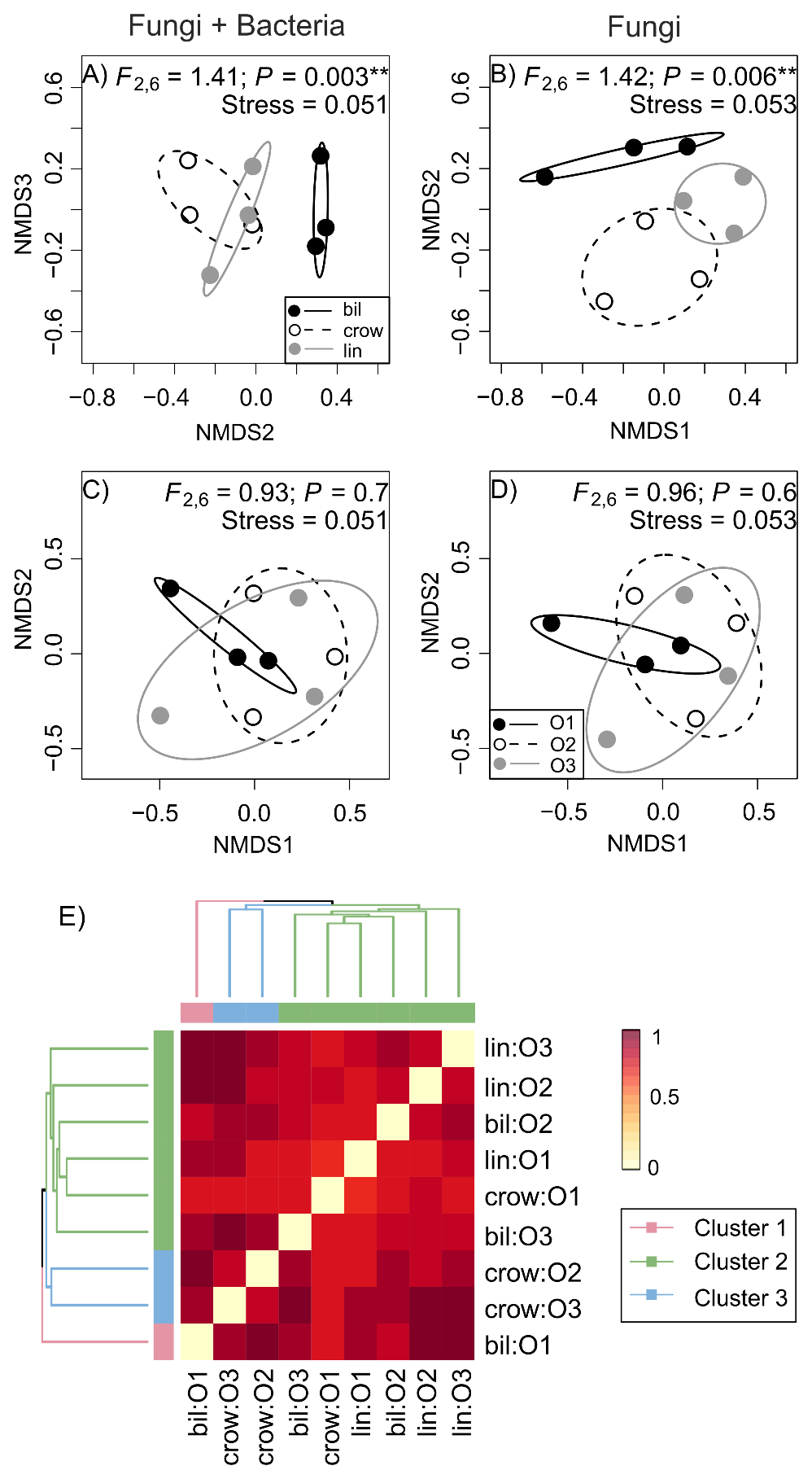


Figure S5: Nonmetric multidimensional scaling (NMDS) ordinations of endophytic community compositions grouped by berry species (A-B), growth sites (C-D), and an UPGMA tree (E) representing the similarity of the endophytic diversity of the samples based on Sørensen dissimilarity matrix. (A-D) Ellipses denote 95% confidence intervals around the group centroid based on standard errors. NMDS axes were chosen to clearest present the grouping. (*) indicates the significance in the statistical tests. Abbreviations: bil - bilberry, crow – crowberry, lin – lingonberry, and growth sites (O1, O2, O3).


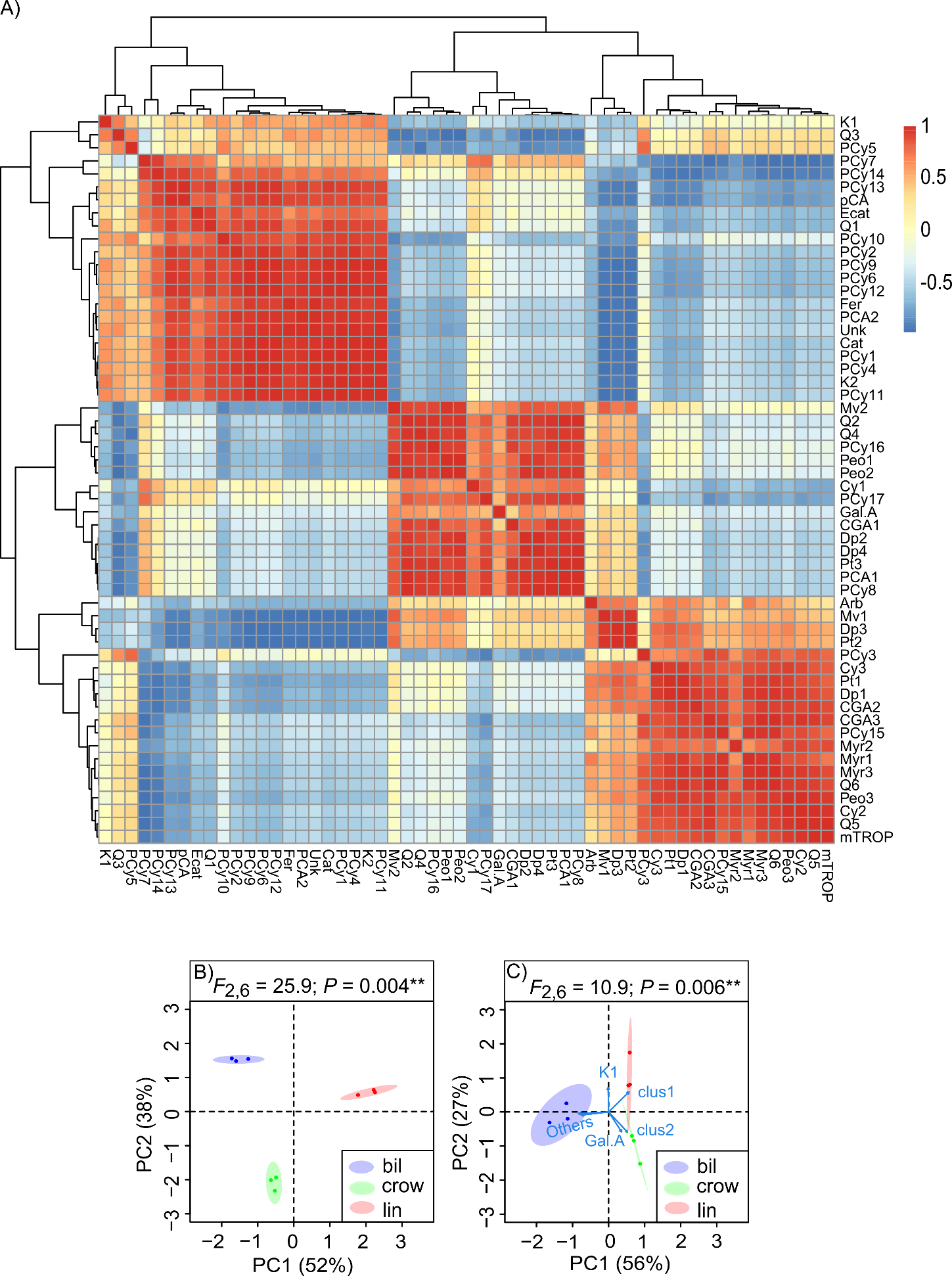


Figure S6: Heatmap of pairwise correlation of standardized phenolic compound data (A), principal component analysis of the Euclidean distance of standardized phenolic compound data before collinearity removal (B) and after collinearity removal (C). A) Colors of the heatmap indicate the value of pairwise correlation coefficient with blue for low value and red for high value. (B, C) Ellipses denote 95% confidence intervals around the berry group centroid based on standard errors. (**) indicates the significance in the statistical tests. Abbreviations: bil - bilberry, crow – crowberry, lin – lingonberry. Others: myricitrin (Myr1), myricetin derivative 2 (Myr2), quercetin 3-O-glycoside 5 (Q5), p-coumaroyl monotropein derivative (mTROP).
