# Supplementary material for: Host species shape the community structure of culturable endophytes in fruits of wild berry species (Vaccinium myrtillus L., Empetrum nigrum L. and Vaccinium vitis-idaea L.)
Source: FEMS Microbiol Ecol. 2021 Jul 12;97(8):fiab097. doi: 10.1093/femsec/fiab097 (PMC8292141; doi:10.1093/femsec/fiab097)
Supplement: fiab097_Supplement_File [file fiab097_supplement_file.zip › Appendix-S2.docx]

Table S1: Locations and amounts of berries collected in 2018 for phenolic compound analysis. Abbreviation: FW- fresh weight.

|  | Geographic coordinates | | | Bilberry | | Lingonberry | | Crowberry | |
| --- | --- | --- | --- | --- | --- | --- | --- | --- | --- |
| Site | Latitude | Longitude | Vegetation types^a^ | FW (g) | Date of collecting | FW (g) | Date of collecting | FW (g) | Date of collecting |
| O1 | 65.059611 N | 25.461306 E | Industrial or commercial units | 150 | 6-Aug-2018 | 130 | 6-Sep-2018 | 23 | 15-Aug-2018 |
| O2 | 65.067111 N | 25.459111 E | Coniferous forest | 100 | 7-Aug-2018 | 140 | 10-Sep-2018 | 63 | 14-Aug-2018 |
| O3 | 65.057944 N | 25.476806 E | Discontinuous urban fabric | 150 | 7-Aug-2018 | 200 | 10-Sep-2018 | 5 | 14-Aug-2018 |

^a^ Vegetation types of the growth sites were extracted from the CORINE Land Cover 2018, 25 ha dataset using the R package raster, rgdal, and sf. The CORINE shapefile was downloaded from [www.syke.fi](http://www.syke.fi).

Table S2: LC-MS data for phenolic compounds quantification. Abbreviations: pyranoside – galactoside and/or glucoside; RT (min) – retention time.

| Full name | Event | Ionisation Mode | Transition | RT (min) | Full name standard |
| --- | --- | --- | --- | --- | --- |
| cyanidin 3-O- pyranoside | MRM | + | 449→287 | 19.87; 20.99 | cyanidin 3-O- glucoside |
| cyanidin 3-O- arabinoside | MRM | + | 419 →287 | 23.38 | cyanidin 3-O- glucoside |
| delphinidin 3-O- pyranoside | MRM | + | 465→303 | 15.31*; 24.36 | cyanidin 3-O- glucoside |
| delphinidin 3-O- arabinoside | MRM | + | 435→303 | 19.42, 26.37 | cyanidin 3-O- glucoside |
| malvidin 3-O- pyranoside | MRM | + | 493→331 | 25.75* | cyanidin 3-O- glucoside |
| malvidin 3-O- arabinoside | MRM | + | 463→331 | 26.82 | cyanidin 3-O- glucoside |
| peonidin 3-O- pyranoside | MRM | + | 463→301 | 24.39; 25.32 | cyanidin 3-O- glucoside |
| peonidin 3-O- arabinoside | MRM | + | 433→301 | 26.37 | cyanidin 3-O- glucoside |
| petunidin 3-O- pyranoside | MRM | + | 479→317 | 22.47* | cyanidin 3-O- glucoside |
| petunidin 3-O- arabinoside | MRM | + | 449→317 | 24,21 | cyanidin 3-O- glucoside |
| petunidin 3-O- rutinoside | MRM | + | 625→317 | 36.82 | cyanidin 3-O- glucoside |
| catechin | MRM | - | 289→245 | 16.44 | catechin |
| epicatechin | MRM | - | 289→245 | 24.25 | epicatechin |
| procyanidin dimer A | SIM | + | 577,0 | \| 24.54; 28.74; 30.40; \| \| --- \| \| 27.77 \| | procyanidins dimer A |
| procyanidin dimer B | SIM | + | 579,0 | 10.33; 14.31; 18.23; 23.28; 29.56; 28.77 | procyanidins dimer B |
| procyanidin trimer A | SIM | + | 863,0 | 28.57 | procyanidins A2 |
| procyanidin trimer B | SIM | + | 865,0 | 11.98; 17.84; 21.88  23.08; 24.30; 28.64 | procyanidins B2 |
| kaempferol 3-O-pyranoside | MRM | - | 447→283 | 36.32; 36.65 | kaempferol 3-O- glucoside |
| quercetin 3-O- pyranoside | MRM | - | 463→301 | 31.74; 32.45; 34.58; 34.88; 36.41; 37.39 | hyperoside |
| myricetin 3-O-rhamnoside | MRM | - | 463,1→316 | 34.44; 36.05 | hyperoside |
| gallic acid | MRM | - | 169,1→151,1 | 8.03 | gallic acid |
| ferulic acid | MRM | - | 193,2→134,2; 193,20→178,25 | 36.07 | ferulic acid |
| caffeoylquinic acid (chlorogenic acid) | MRM | - | 353,1→191,1 | 12.86; 20.70**; 25.34 | 3-O-caffeoylquinic acid |
| protocatechuic acid | MRM | - | 153→108; | 12.97; 19.88 | protocatechuic acid |
| arbutin | MRM | - | 271,1→161,1 | 38.75 | catechin |
| *p*-coumaric acid | MRM | - | 163,10 → 119,10 | 30.97 | *p*-coumaric acid |
| *p*-coumaroyl monotropein derivative *** | UV |  |  | 33.37 | *p*-coumaric acid |

*: pyranoside (galactoside and/or glucoside).

**: 3-O-caffeoylquinic acid

***: *p*-coumaroyl monotropein derivative (Heffels et al. 2017, Food Res Int, 100: 462-468, DOI : 10.1016/j.foodres.2016.11.018). Identification : lmax = 311nm, [M-H] = 535, [M+formate-H] = 581. UV quantification at 320nm.
