# Supplementary material for: Host species shape the community structure of culturable endophytes in fruits of wild berry species (Vaccinium myrtillus L., Empetrum nigrum L. and Vaccinium vitis-idaea L.)
Source: FEMS Microbiol Ecol. 2021 Jul 12;97(8):fiab097. doi: 10.1093/femsec/fiab097 (PMC8292141; doi:10.1093/femsec/fiab097)
Supplement: fiab097_Supplement_File [file fiab097_supplement_file.zip › Appendix-S5.docx]

Table S5: Partitioned beta diversity metrics for endophytic community composition within each host and each growth site.

| *Host* | *Bilberry* | | | *Crowberry* | | | *Lingonberry* | | |
| --- | --- | --- | --- | --- | --- | --- | --- | --- | --- |
|  | *Fungi + Bacteria* | *Fungi* |  | *Fungi + Bacteria* | *Fungi* |  | *Fungi + Bacteria* | *Fungi* |  |
| Turnover (β_SIM_) | 0.854 | 0.857 |  | 0.692 | 0.650 |  | 0.797 | 0.793 |  |
| Nestedness (β_SNE_) | 0.011 | 0.013 |  | 0.122 | 0.153 |  | 0.032 | 0.025 |  |
| Overall (β_SOR_) | 0.865 | 0.870 |  | 0.814 | 0.803 |  | 0.829 | 0.818 |  |
| *Site* | *O1* | | | *O2* | | | *O3* | | |
|  | *Fungi + Bacteria* | *Fungi* |  | *Fungi + Bacteria* | *Fungi* |  | *Fungi + Bacteria* | *Fungi* |  |
| Turnover (β_SIM_) | 0.768 | 0.745 |  | 0.856 | 0.844 |  | 0.914 | 0.891 |  |
| Nestedness(β_SNE_) | 0.056 | 0.069 |  | 0.026 | 0.026 |  | 0.015 | 0.027 |  |
| Overall (β_SOR_) | 0.824 | 0.814 |  | 0.881 | 0.870 |  | 0.929 | 0.918 |  |

Table S6: Phenolic compounds concentrations (mg/g DW) in bilberry, crowberry, and lingonberry, in August 2018 in three sites in Oulu. Values are mean of three replicates extractions (SD < 8.7% for compounds more abundant than 0.01 mg/g DW and SD < 10.0% if all compounds more abundant than 0.005 mg/g DW are included).

| *Group* | *Compound* | *Compound ID* | *bilberry* | | | *crowberry* | | | *lingonberry* | | |
| --- | --- | --- | --- | --- | --- | --- | --- | --- | --- | --- | --- |
|  |  |  | *site O1* | *site O2* | *site O3* | *site O1* | *site O2* | *site O3* | *site O1* | *site O2* | *site O3* |
| anthocyanin | cyanidin 3-O-galactoside | Cy1 | 5.69 | 6.06 | 4.14 | 7.83 | 8.92 | 9.66 | 6.63 | 5.55 | 8.41 |
| anthocyanin | cyanidin 3-O-glucoside | Cy2 | 5.64 | 4.34 | 4.28 | 0.30 | 0.32 | 0.31 | 0.51 | 0.28 | 0.77 |
| anthocyanin | cyanidin 3-O-arabinoside | Cy3 | 4.84 | 4.72 | 3.57 | 2.01 | 2.74 | 2.39 | 1.78 | 1.14 | 2.47 |
| anthocyanin | delphinidin 3-O-arabinoside | Dp1 | 1.93 | 2.41 | 1.54 | 0.28 | 0.48 | 0.46 | 0.00 | 0.00 | 0.00 |
| anthocyanin | delphinidin 3-O-arabinoside 2 | Dp2 | nd | nd | nd | 0.06 | 0.06 | 0.05 | nd | nd | nd |
| anthocyanin | delphinidin 3-O-pyroside | Dp3 | 4.10 | 4.81 | 3.28 | 2.18 | 3.75 | 3.79 | 0.01 | 0.00 | 0.00 |
| anthocyanin | delphinidin 3-O-pyroside 2 | Dp4 | nd | nd | nd | 0.15 | 0.13 | 0.13 | nd | nd | nd |
| anthocyanin | malvidin 3-O-arabinoside | Mv1 | 1.16 | 1.31 | 0.69 | 0.97 | 1.15 | 1.00 | 0.00 | 0.00 | 0.00 |
| anthocyanin | malvidin 3-O-pyroside | Mv2 | 3.59 | 3.83 | 2.65 | 6.47 | 7.60 | 6.80 | 0.01 | 0.00 | 0.00 |
| anthocyanin | peonidin 3-O-arabinoside | Peo1 | 0.75 | 0.64 | 0.46 | 2.34 | 2.15 | 1.99 | 0.00 | 0.00 | 0.00 |
| anthocyanin | peonidin 3-O-galactoside | Peo2 | 1.38 | 1.29 | 0.91 | 5.54 | 5.19 | 5.09 | 0.01 | 0.01 | 0.01 |
| anthocyanin | peonidin 3-O-glucoside | Peo3 | 3.14 | 2.39 | 2.28 | 0.31 | 0.27 | 0.31 | 0.00 | 0.01 | 0.01 |
| anthocyanin | petunidin 3-O-arabinoside | Pt1 | 0.91 | 1.11 | 0.67 | 0.19 | 0.31 | 0.29 | 0.00 | 0.00 | 0.00 |
| anthocyanin | petunidin 3-O-pyroside | Pt2 | 2.74 | 3.19 | 2.24 | 1.72 | 2.80 | 2.61 | 0.00 | 0.00 | 0.00 |
| anthocyanin | petunidin 3-O-rutinoside | Pt3 | nd | nd | nd | 0.09 | 0.07 | 0.12 | nd | nd | nd |
| flavan-3-ols | epicatechin | Ecat | 0.23 | 0.26 | 0.24 | 0.26 | 0.27 | 0.34 | 0.43 | 0.30 | 0.38 |
| flavan-3-ols | catechin | Cat | 0.01 | 0.01 | 0.01 | 0.11 | 0.12 | 0.10 | 1.15 | 0.96 | 1.34 |
| Phenolic acids | gallic acid | Gal.A | nd | nd | nd | 0.03 | 0.18 | 0.03 | nd | nd | nd |
| Phenolic acids | protocatechuic acid derivative | PCA1 | 0.02 | 0.03 | 0.04 | 0.59 | 0.64 | 0.80 | 0.03 | 0.03 | 0.05 |
| Phenolic acids | protocatechuic acid derivative 2 | PCA2 | nd | nd | nd | nd | nd | nd | 0.06 | 0.05 | 0.04 |
| Phenolic acids | chlorogenic acid 1 | CGA1 | nd | nd | nd | 0.34 | 0.28 | 0.79 | nd | nd | nd |
| Phenolic acids | chlorogenic acid 2 | CGA2 | 2.18 | 2.44 | 2.57 | 0.47 | 0.43 | 1.15 | 0.15 | 0.15 | 0.30 |
| Phenolic acids | chlorogenic acid 3 | CGA3 | 0.12 | 0.13 | 0.12 | nd | nd | nd | 0.02 | 0.01 | 0.03 |
| Phenolic acids | ferulic derivative | Fer | nd | nd | nd | nd | nd | nd | 0.17 | 0.24 | 0.19 |
| Phenolic acids | *p*-coumaric acid | pCA | 0.03 | 0.02 | 0.04 | 0.14 | 0.17 | 0.24 | 0.44 | 0.46 | 0.39 |
| Flavonols | myricitrin | Myr1 | 0.02 | 0.03 | 0.02 | nd | nd | nd | nd | nd | nd |
| Flavonols | myricetin derivative 2 | Myr2 | 0.00 | 0.00 | 0.00 | nd | nd | nd | nd | nd | nd |
| Flavonols | myricetin derivative 3 | Myr3 | 0.00 | 0.00 | 0.00 | nd | nd | nd | nd | nd | nd |
| Flavonols | quercetin 3-O-glycoside 1 | Q1 | 0.14 | 0.16 | 0.11 | 0.24 | 0.18 | 0.32 | 0.56 | 0.34 | 0.62 |
| Flavonols | quercetin 3-O-glycoside 2 | Q2 | 0.19 | 0.17 | 0.18 | 0.71 | 0.79 | 0.99 | 0.07 | 0.04 | 0.09 |
| Flavonols | quercetin 3-O-glycoside 3 | Q3 | 0.43 | 0.51 | 0.66 | nd | nd | nd | 0.44 | 0.79 | 0.73 |
| Flavonols | quercetin 3-O-glycoside 4 | Q4 | 0.29 | 0.27 | 0.25 | 0.44 | 0.47 | 0.53 | 0.16 | 0.26 | 0.25 |
| Flavonols | quercetin 3-O-glycoside 5 | Q5 | 0.01 | 0.01 | 0.01 | nd | nd | nd | nd | nd | nd |
| Flavonols | quercetin 3-O-glycoside 6 | Q6 | 0.01 | 0.01 | 0.01 | nd | nd | nd | nd | nd | nd |
| Flavonols | kaempferol 3-O-galactoside | K1 | 0.01 | 0.01 | 0.01 | 0.00 | 0.00 | 0.00 | 0.01 | 0.01 | 0.02 |
| Flavonols | kaempferol 3-O-glucoside | K2 | 0.01 | 0.01 | 0.01 | 0.01 | 0.01 | 0.01 | 0.12 | 0.10 | 0.16 |
| Other | arbutin | Arb | 0.00 | 0.01 | 0.00 | 0.00 | 0.00 | 0.01 | 0.00 | 0.00 | 0.00 |
| Other | unknown | Unk | 0.01 | 0.01 | 0.01 | nd | nd | nd | 0.18 | 0.19 | 0.20 |
| Other | *p*-coumaroyl monotropein derivative | mTROP | 1.06 | 0.54 | 0.97 | nd | nd | nd | nd | nd | nd |
| Proanthocyanidins | procyanidin dimer B | PCy1 | 0.02 | 0.03 | 0.03 | 0.12 | 0.13 | 0.15 | 3.77 | 3.12 | 4.16 |
| Proanthocyanidins | procyanidin dimer B | PCy2 | 0.04 | 0.04 | 0.04 | 0.10 | 0.12 | 0.13 | 0.86 | 0.49 | 0.59 |
| Proanthocyanidins | procyanidin dimer B (B2) | PCy3 | 0.90 | 1.02 | 1.08 | 0.22 | 0.23 | 0.35 | 0.72 | 0.55 | 0.57 |
| Proanthocyanidins | procyanidin dimer B | PCy4 | nd | nd | nd | 0.01 | 0.01 | 0.02 | 0.33 | 0.25 | 0.34 |
| Proanthocyanidins | procyanidin dimer B | PCy5 | 0.13 | 0.15 | 0.16 | 0.09 | 0.08 | 0.10 | 0.19 | 0.13 | 0.14 |
| Proanthocyanidins | procyanidin dimer A | PCy6 | nd | nd | nd | 0.22 | 0.22 | 0.34 | 1.49 | 1.23 | 1.52 |
| Proanthocyanidins | procyanidin dimer A (A2) | PCy7 | 0.02 | 0.01 | 0.02 | 1.46 | 1.44 | 1.99 | 1.97 | 1.30 | 1.49 |
| Proanthocyanidins | procyanidin trimer A | PCy8 | 0.00 | 0.00 | 0.00 | 0.12 | 0.11 | 0.14 | 0.01 | 0.00 | 0.00 |
| Proanthocyanidins | procyanidin trimer B | PCy9 | nd | nd | nd | 0.01 | 0.01 | 0.01 | 0.08 | 0.07 | 0.10 |
| Proanthocyanidins | procyanidin trimer B | PCy10 | 0.18 | 0.18 | 0.12 | 0.10 | 0.10 | 0.08 | 0.47 | 0.27 | 0.41 |
| Proanthocyanidins | procyanidin trimer B | PCy11 | nd | nd | nd | nd | nd | nd | 0.05 | 0.05 | 0.06 |
| Proanthocyanidins | procyanidin trimer B | PCy12 | nd | nd | nd | 0.05 | 0.05 | 0.08 | 0.33 | 0.28 | 0.35 |
| Proanthocyanidins | procyanidin trimer B | PCy13 | nd | nd | nd | 0.12 | 0.13 | 0.17 | 0.44 | 0.34 | 0.38 |
| Proanthocyanidins | procyanidin trimer B | PCy14 | 0.00 | 0.00 | 0.00 | 0.13 | 0.13 | 0.18 | 0.27 | 0.20 | 0.20 |
| Proanthocyanidins | procyanidin dimer A | PCy15 | 0.04 | 0.07 | 0.05 | nd | nd | nd | 0.01 | 0.00 | 0.01 |
| Proanthocyanidins | procyanidin dimer A | PCy16 | 0.01 | 0.01 | 0.02 | 0.04 | 0.04 | 0.06 | nd | nd | nd |
| Proanthocyanidins | procyanidin dimer B | PCy17 | 0.01 | 0.01 | 0.02 | 0.44 | 0.34 | 0.51 | 0.20 | 0.13 | 0.15 |

Abbreviation: nd: not detected

Table S7: Summary of the phenolic composition of the different berry types in each site, presented as groups of compounds and expressed as mg/g DW and as % of the total amount of soluble phenolics quantified.

| *Berry* |  | *Bilberry* | | | *Crowberry* | | | *Lingonberry* | | |
| --- | --- | --- | --- | --- | --- | --- | --- | --- | --- | --- |
| *Site* |  | *O1* | *O2* | *O3* | *O1* | *O2* | *O3* | *O1* | *O2* | *O3* |
| mg/g DW | anthocyanin | 35.87 | 36.10 | 26.72 | 30.43 | 35.92 | 34.98 | 8.95 | 6.98 | 11.68 |
|  | flavan-3-ols | 0.24 | 0.27 | 0.25 | 0.37 | 0.38 | 0.44 | 1.58 | 1.26 | 1.71 |
|  | Flavonols | 1.11 | 1.17 | 1.25 | 1.40 | 1.45 | 1.86 | 1.34 | 1.53 | 1.86 |
|  | Other | 1.07 | 0.56 | 0.99 | 0.01 | 0.00 | 0.01 | 0.18 | 0.19 | 0.20 |
|  | Phenolic.acids | 2.35 | 2.61 | 2.77 | 1.57 | 1.69 | 3.01 | 0.86 | 0.94 | 1.01 |
|  | Proanthocyanidins | 1.37 | 1.53 | 1.53 | 3.25 | 3.14 | 4.32 | 11.19 | 8.41 | 10.49 |
|  | Total | 42.01 | 42.24 | 33.51 | 37.02 | 42.59 | 44.61 | 24.10 | 19.31 | 26.95 |
|  |  |  |  |  |  |  |  |  |  |  |
| % | anthocyanin | 85.39 | 85.45 | 79.72 | 82.20 | 84.33 | 78.42 | 37.15 | 36.12 | 43.32 |
|  | flavan-3-ols | 0.56 | 0.63 | 0.74 | 1.00 | 0.90 | 0.99 | 6.56 | 6.53 | 6.36 |
|  | Flavonols | 2.65 | 2.78 | 3.74 | 3.77 | 3.41 | 4.16 | 5.57 | 7.95 | 6.90 |
|  | Other | 2.56 | 1.32 | 2.95 | 0.01 | 0.01 | 0.01 | 0.74 | 1.01 | 0.75 |
|  | Phenolic.acids | 5.59 | 6.19 | 8.27 | 4.23 | 3.96 | 6.74 | 3.56 | 4.86 | 3.74 |
|  | Proanthocyanidins | 3.26 | 3.63 | 4.58 | 8.79 | 7.38 | 9.68 | 46.42 | 43.54 | 38.93 |
|  | Total | 100 | 100 | 100 | 100 | 100 | 100 | 100 | 100 | 100 |

Table S8: New matrix of phenolic data after removal of collinearity and standardization. Clus1 and clus2 are the two clusters after collinearity removal. The remaining variables are phenolic variables which did not belong to any cluster when using the threshold 0.43.

| Samples | clus1^a^ | clus2^b^ | Gal.A^c^ | Myr1^c^ | Myr2^c^ | Q5^c^ | K1^c^ | mTROP^c^ |
| --- | --- | --- | --- | --- | --- | --- | --- | --- |
| BO1 | -0.97 | -0.67 | -0.46 | 0.80 | 2.41 | 1.58 | -0.34 | 1.72 |
| BO2 | -1.12 | -0.77 | -0.46 | 2.16 | 0.44 | 0.85 | -0.26 | 0.57 |
| BO3 | -0.83 | -0.86 | -0.46 | 0.69 | 0.44 | 1.49 | 0.56 | 1.52 |
| CO1 | -0.27 | 1.22 | 0.05 | -0.61 | -0.55 | -0.65 | -0.75 | -0.63 |
| CO2 | -0.36 | 1.19 | 2.59 | -0.61 | -0.55 | -0.65 | -0.75 | -0.63 |
| CO3 | -0.26 | 1.55 | 0.14 | -0.61 | -0.55 | -0.65 | -0.78 | -0.63 |
| LO1 | 1.39 | -0.59 | -0.46 | -0.61 | -0.55 | -0.65 | -0.15 | -0.63 |
| LO2 | 1.07 | -0.56 | -0.46 | -0.61 | -0.55 | -0.65 | 0.07 | -0.63 |
| LO3 | 1.34 | -0.50 | -0.46 | -0.61 | -0.55 | -0.65 | 2.40 | -0.63 |

Abbreviations: berry species (B: bilberry, C: crowberry, L: lingonberry), growth sites (O1, O2, O3)

^a^clus1 is the collinearity-removal cluster score from the combination of cyanidin 3-O-arabinoside (Cy3) , delphinidin 3-O-arabinoside (Dp1), delphinidin 3-O-pyroside (Dp3), malvidin 3-O-arabinoside (Mv1), peonidin 3-O-glucoside (Peo3), petunidin 3-O-arabinoside (Pt1), petunidin 3-O-pyroside (Pt2), epicatechin (Ecat), catechin (Cat), protocatechuic acid derivative 2 (PCA2), chlorogenic acid 2 (CGA2), ferulic derivative (Fer), myricetin derivative 3 (Myr3), quercetin 3-O-glycoside (Q1, Q6), kaempferol 3-O-glucoside (K2), arbutin (Arb), unknown (Unk), procyanidin dimer B (PCy1, PCy2, PCy4), procyanidin dimer A (PCy6), procyanidin trimer B (PCy9, PCy10, PCy11, PCy12, PCy13, PCy14), *p*-coumaric acid (pCA).

^b^clus2 is the collinearity-removal cluster score from the combination of cyanidin 3-O-galactoside (Cy1), cyanidin 3-O-glucoside (Cy2), delphinidin 3-O-arabinoside 2 (Dp2), delphinidin 3-O-pyroside 2 (Dp4), malvidin 3-O-pyroside (Mv2), peonidin 3-O-arabinoside (Peo1), peonidin 3-O-galactoside (Peo2), petunidin 3-O-rutinoside (Pt3), protocatechuic acid derivative (PCA1), chlorogenic acid (CGA, CGA3), quercetin 3-O-glycoside (Q2, Q3, Q4), procyanidin dimer B (PCy3, PCy5, PCy17), procyanidin dimer A (PCy7, PCy15, PCy16), procyanidin trimer A (PCy8).

^c^ unclustered variables: gallic acid (Gal.A), myricitrin (Myr1), myricetin derivative 2 (Myr2), quercetin 3-O-glycoside 5 (Q5), kaempferol 3-O-galactoside (K1), *p*-coumaroyl monotropein derivative (mTROP)
