# Supplementary material for: Host species shape the community structure of culturable endophytes in fruits of wild berry species (Vaccinium myrtillus L., Empetrum nigrum L. and Vaccinium vitis-idaea L.)
Source: FEMS Microbiol Ecol. 2021 Jul 12;97(8):fiab097. doi: 10.1093/femsec/fiab097 (PMC8292141; doi:10.1093/femsec/fiab097)
Supplement: fiab097_Supplement_File [file fiab097_supplement_file.zip › Revised-Supporting-Information-captions.docx]

**Supporting information**

Additional Supporting Information may be found in the online version of this article at the publisher’s website:

**Appendix S1 includes the following supplementary figures:**

Figure S1: Sampling sites in Oulu, Finland (O1: 65.059611 N 25.461306 E; O2: 65.067111 N 25.459111 E; O3: 65.057944 N 25.476806 E). Each site contains three berry species: bilberry, crowberry, and lingonberry. The distance between sampling sites: O1-O2: 750 m; O1-O3: 400 m; O2-O3: 950 m. Red squares indicate the area where the samples were collected.

Figure S2: Fungal endophytes of bilberry from three growth sites. The species were marked by their ID (e.g. E1) and grouped by their closest ancestors (e.g. Cladosporium sp.).

Figure S3: Fungal endophytes of crowberry from three growth sites. The species were marked by their ID (e.g. E1) and grouped by their closest ancestors (e.g. Cladosporium sp.).

Figure S4: Fungal endophytes of lingonberry from three growth sites. The species were marked by their ID (e.g. E1) and grouped by their closest ancestors (e.g. Cladosporium sp.).

Figure S5: Nonmetric multidimensional scaling (NMDS) ordinations of endophytic community compositions grouped by berry species (A-B), growth sites (C-D), and an UPGMA tree (E) representing the similarity of the endophytic diversity of the samples based on Sørensen dissimilarity matrix. (A-D) Ellipses denote 95% confidence intervals around the group centroid based on standard errors. NMDS axes were chosen to clearest present the grouping. (*) indicates the significance in the statistical tests. Abbreviations: bil - bilberry, crow – crowberry, lin – lingonberry, and growth sites (O1, O2, O3).

Figure S6: Heatmap of pairwise correlation of standardized phenolic compound data (A), principal component analysis of the Euclidean distance of standardized phenolic compound data before collinearity removal (B) and after collinearity removal (C). A) Colors of the heatmap indicate the value of pairwise correlation coefficient with blue for low value and red for high value. (B, C) Ellipses denote 95% confidence intervals around the berry group centroid based on standard errors. (**) indicates the significance in the statistical tests. Abbreviations: bil - bilberry, crow – crowberry, lin – lingonberry. Others: myricitrin (Myr1), myricetin derivative 2 (Myr2), quercetin 3-O-glycoside 5 (Q5), p-coumaroyl monotropein derivative (mTROP).

**Appendix S2 includes the following supplementary tables:**

Table S1: Locations and amounts of berries collected in 2018 for phenolic compound analysis. Abbreviation: FW- fresh weight.

Table S2: LC-MS data for phenolic compounds quantification. Abbreviations: pyranoside – galactoside and/or glucoside; RT (min) – retention time.

**Appendix S3 includes the following supplementary table:**

Table S3: Proportions of fungal endophytic taxa (%) and the average proportion across all samples for each taxon were shown. Abbreviations: kingdom (k), phylum (p), class (c), order (o), family (f) and genus (g).

**Appendix S4 includes the following supplementary table:**

Table S4: The number of bacteria isolated from each sample for each taxon was shown. Abbreviations: kingdom (k), phylum (p), class (c), order (o), family (f) and genus (g). We did not calculate the proportion (%) for each taxon because of the absence of bacteria in lingonberry at site O3 and the low quantity of the isolated bacteria.

**Appendix S5 includes the following supplementary tables:**

Table S5: Partitioned beta diversity metrics for endophytic community composition within each host and each growth site.

Table S6: Phenolic compounds concentrations (mg/g DW) in bilberry, crowberry, and lingonberry, in August 2018 in three sites in Oulu. Values are mean of three replicates extractions (SD < 8.7% for compounds more abundant than 0.01 mg/g DW and SD < 10.0% if all compounds more abundant than 0.005 mg/g DW are included).

Table S7: Summary of the phenolic composition of the different berry types in each site, presented as groups of compounds and expressed as mg/g DW and as % of the total amount of soluble phenolics quantified.

Table S8: New matrix of phenolic data after removal of collinearity and standardization. Clus1 and clus2 are the two clusters after collinearity removal. The remaining variables are phenolic variables which did not belong to any cluster when using the threshold 0.43.
